# Insight into the Role and Regulation of Gap Junction Genes in Lung Cancer and Identification of Nuclear Cx43 as a Putative Biomarker of Poor Prognosis

**DOI:** 10.3390/cancers11030320

**Published:** 2019-03-06

**Authors:** Trond Aasen, Irene Sansano, Maria Ángeles Montero, Cleofé Romagosa, Jordi Temprana-Salvador, Alexandre Martínez-Marti, Teresa Moliné, Javier Hernández-Losa, Santiago Ramón y Cajal

**Affiliations:** 1 Translational Molecular Pathology, Vall d’Hebron Research Institute (VHIR), 08035 Barcelona, Spain; sramon@vhebron.net; 2 Pathology Department, Vall d’Hebron University Hospital, 08035 Barcelona, Spain; isansava@gmail.com (I.S.); angeles.montero@mft.nhs.uk (M.Á.M.); cleoferoma@gmail.com (C.R.); jordi.temprana@gmail.com (J.T.-S.); teresa.moline@vhir.org (T.M.); jahernan@vhebron.net (J.H.-L.); 3 Medical Oncology Department, Vall d’Hebron University Hospital, 08035 Barcelona, Spain; alexmartinezmarti.oncologia@gmail.com

**Keywords:** connexins, Cx43, gap junctions, lung cancer, immunohistochemistry, prognosis, nuclear

## Abstract

Direct intercellular communication, mediated by gap junctions formed by the connexin transmembrane protein family, is frequently dysregulated in cancer. Connexins have been described as tumour suppressors, but emerging evidence suggests that they can also act as tumour promoters. This feature is connexin- and tissue-specific and may be mediated by complex signalling pathways through gap junctions or hemichannels or by completely junction-independent events. Lung cancer is the number one cancer in terms of mortality worldwide, and novel biomarkers and therapeutic targets are urgently needed. Our objective was to gain a better understanding of connexins in this setting. We used several in silico tools to analyse TCGA data in order to compare connexin mRNA expression between healthy lung tissue and lung tumours and correlated these results with gene methylation patterns. Using Kaplan-Meier plotter tools, we analysed a microarray dataset and an RNA-seq dataset of non-small cell lung tumours in order to correlate connexin expression with patient prognosis. We found that connexin mRNA expression is frequently either upregulated or downregulated in lung tumours. This correlated with both good and poor prognosis (overall survival) in a clear connexin isoform-dependent manner. These associations were strongly influenced by the histological subtype (adenocarcinoma versus squamous cell carcinoma). We present an overview of all connexins but particularly focus on four isoforms implicated in lung cancer: Cx26, Cx30.3, Cx32 and Cx43. We further analysed the protein expression and localization of Cx43 in a series of 73 human lung tumours. We identified a subset of tumours that exhibited a unique strong nuclear Cx43 expression pattern that predicted worse overall survival (*p* = 0.014). Upon sub-stratification, the prognostic value remained highly significant in the adenocarcinoma subtype (*p* = 0.002) but not in the squamous carcinoma subtype (*p* = 0.578). This finding highlights the importance of analysis of connexin expression at the protein level, particularly the subcellular localization. Elucidation of the underlying pathways regulating Cx43 localization may provide for novel therapeutic opportunities.

## 1. Introduction

The World Health Organization (WHO) currently estimates that 1.69 million deaths per year worldwide are due to lung cancer, far more than any other cancer type. Non-small cell lung cancer (NSCLC), which includes the two major subtypes of lung adenocarcinoma (LUAD) and lung squamous cell carcinoma (LUSC), accounts for about 85% of lung cancers. In terms of the biology of lung cancer, significant advances have been made [1]. Indeed, LUAD and LUSC has been shown to originate from different cells and have unique molecular characteristics leading to distinct biological and pathological patterns needing different therapeutic strategies. The delineation of specific oncogenic pathways has allowed stratification of tumours and incorporation of patient-specific targeted therapy based on, for example, the activation status of receptor tyrosine kinases such as epidermal growth factor receptor (EGFR) [1]. Nevertheless, lung cancer is a highly heterogeneous disease at both the histological and molecular levels, and metastasis is frequently present at diagnosis, which together result in a poor overall survival (OS) rate [1]. Thus, identification of additional biomarkers, elucidation of the full oncogenic circuit defining these tumours and novel treatment approaches are some of the steps needed for the future management of this disease.

Lung tissue homeostasis is thought to be tightly controlled through multiple mechanisms, including direct gap junction-mediated intercellular communication (GJIC). Gap junctions are formed by transmembrane proteins named connexins that assemble into hexameric structures called connexons, which act as hemichannels that can dock with hemichannels in adjacent cells forming an intercellular channel. The 21-member family of human connexin proteins ranges in size between 23 and 62 kDa (Cx23-Cx62) [2]. It is also thought that hemichannels may additionally communicate with the extracellular matrix under specific pathological conditions such as cancer [3]. Moreover, several connexins (most notably Cx43) have been shown to possess functions unrelated to the formation of junctional complexes [4,5]. *GJA1*, which encodes Cx43, has been shown to synthesize several truncated protein forms through a process of internal translation initiation regulated by key cancer signalling pathways such as mTOR and Mnk1/2 [6,7,8]. The role of these protein forms is currently being explored but includes an interaction with the cytoskeleton and mitochondria [9,10,11] and the regulation of epithelial to mesenchymal transition (EMT) [12,13].

A variety of connexins are expressed in lung tissue and are thought to be critical for lung physiology, function and host defences under normal and pathological conditions [14]. Cx26 and Cx43 have been detected in human epithelial cells of the respiratory airways, whereas a variety of additional connexins has been detected in murine models, including Cx32, Cx37 and Cx46 [14]. Targeting of connexins has been proposed as a viable approach in terms of the management of lung diseases such as cancer [15]. The importance of connexins in cancer in general has been widely studied in vitro and in vivo for more than 50 years, with the work revealing extensive tissue- and connexin-dependent variations [16].

Several studies have shown that connexins may play an important role in lung cancer. Some in vitro studies have suggested a tumour suppressor role for connexins in the lung. Notably, Cx43 gene transfection can inhibit the migration of the human lung squamous carcinoma cell line NCI-H226 [17]. Other work showed that Cx43 may recruit E-cadherin to inhibit the malignant behaviour of lung cancer cells [18]. Cx43 also suppresses lung cancer cell invasion and metastasis, possibly by acting as a “histone deacetylase inhibitor” affecting the gene expression of several genes, such as by increasing the expression and secretion of FSTL1 (follistatin-like 1) [19]. A recent study demonstrated that overexpression of Cx43 in lung cancer cell lines represses cancer stem cells and associated malignant features [20]. Downregulation of Cx26 expression has been shown to occur in several human lung cancer cell lines due to gene promoter methylation [21]. Cx31.1 is also downregulated in NSCLC cell lines, and Cx31.1 re-expression inhibits cell proliferation and metastasis [22]. Degradation of Cx31.1 in lung cancer may also involve clathrin-mediated autophagy [23].

Connexins can also influence the effect of chemotherapy. In A549 LUAD cells, Cx32 increases vinorelbine-induced cytotoxicity by reducing the expression of the multidrug resistance-1 (MDR-1) gene [24]. Other major pathways regulated by Cx32 in lung cancer cells include inhibition of Src activity [25].

Murine models support the link between connexins and lung cancer [16]. Notably, Cx32 knockout mice exhibit a significantly increased incidence of chemical and radiation-induced lung tumours [26,27], likely in part due to activation of the MAPK pathway. Surprisingly, these mice do not show obvious pulmonary alterations but are susceptible to benzene-induced lung toxicity [28]. Cx32-deficient mice seem to display increased proliferation of non-tumoral alveolar epithelial type II (ATII), from which lung tumours originate [26,27,28]. Cx43 heterozygote knockout mice also display increased cell proliferation of ATII cells [29]. A higher incidence of LUADs induced by DMBA is seen in this model [30]. Paradoxically, however, despite higher susceptibility of spontaneous and NNK-induced lung neoplasms, Cx43 mRNA is significantly increased during tumour progression and correlates with increased tumour aggressiveness [31].

In addition, a number of reports have shown altered connexin expression in human tumours. Various studies have suggested promotor methylation regulate connexin expression in cancer [32,33]. Decreased expression of Cx43 at the mRNA and protein levels due to promoter methylation has been shown to occur during NSCLC tumour progression [34]. Hypermethylation of the *GJA1* (Cx43) promoter has been significantly associated with heavy smoking, poorly differentiated NSCLC and low expression of Cx43 [35]. There is also strong evidence that promoter methylation can cause decreased Cx26 expression in lung tumours [21]. Cx32 has been positively correlated with the degree of tumour differentiation and survival rates of NSCLC patients [36]. However, Cx26 was not correlated with smoking, tumour size, histological type, the degree of differentiation, lymph node metastasis and the postoperative survival time [36]. More recently, expression of Cx43 at the time of diagnosis was shown to predict survival in advanced NSCLC patients treated with cisplatin-based chemotherapy [37].

However, although connexins have typically been classified as tumour suppressors, accumulating data suggest that connexins can also promote tumour progression in certain tissues and at certain cancer stages [16,38,39]. This dichotomy is also observed in lung cancer, both in vitro and in vivo, which again depends on the connexin isoform. For example, Cx43 reverses cisplatin resistance in A549 LUAD cells by inhibiting EMT [40]. In contrast, Cx26 (in a GJIC-independent manner) induces EMT via the PI3K-AKT signalling pathway and confers resistance to the EGFR inhibitor gefitinib in HCC827 and PC9 LUAD cells [41]. Indeed, increased expression of Cx26 at the invasive front of LUSC was shown to be significantly correlated with poor prognosis [42]. Other studies have implicated Cx43 in this process through enhanced attachment of lung tumour cells to the endothelium that facilitates extravasation, a critical feature for efficient metastasis [43]. Recently, a clear pro-tumorigenic role for Cx43 in lung cancer and metastasis to the brain was demonstrated. In a series of elegant experiments, Massagué and colleagues [44] showed that lung (and breast) carcinoma cells upregulate Cx43 expression and establish heterocellular GJIC with astrocytes in the brain, creating a cell signalling feedback loop that fosters tumour growth and chemoresistance. FDA-approved compounds that block GJIC significantly inhibit metastasis growth. Others have shown that GJIC-mediated transfer of small RNAs from lung cancer cells to astrocytes can alter cancer cell resistance to chemotherapy [45]. More recently, Cx30.3 was shown to be overexpressed in lung tumours and to be associated with poor prognosis and recurrence [46]. Functionally, Cx30.3 appears to activate the c-Src proto-oncogene to induce a number of cellular traits associated with malignancy.

Thus, there are highly conflicting reports with regards to the pro-tumorigenic and anti-tumorigenic functions of connexins in lung cancer. In addition to GJIC-mediated mechanisms, non-junctional connexin functions must also be carefully considered in lung cancer. Notably, the role of nuclear Cx43 remains to be explored. Nuclear Cx43 has been described in some human tumours including glioma [47] and colon tumours [48]. Nuclear Cx43 was described in lung cancer cell lines more than 20 years ago [49]. Indeed, overexpression of oncogenes such as c-Src and Her2 (frequently observed in the lung) was shown to correlate with nuclear Cx43 localization in rat liver epithelial cells [50]. More recently, Cx43 was shown to be translocated to the nucleus in late G1 of the cell cycle via binding to A-kinase anchoring protein 95 in lung A549 adenocarcinoma cells [51]. Nuclear localization of a truncated form of Cx43 has also been described in a glioma cell line [52]. This is of interest in relation to the recent discovery of the active translation of truncated Cx43 isoforms (notably the GJA1-20k form) [6,7,8,33], which we showed also occur in human lung tumour cell lines under regulation of oncogenic pathways frequently activated in lung cancer such as mTOR and MAPK-Mnk1/2 kinases [7]. Very recently, the major truncated form of Cx43 (GJA1-20k) was shown to regulate EMT by acting as a direct nuclear transcriptional activator of N-cadherin [13].

To answer some of the discrepancies and outstanding questions discussed above, we have analysed the gene expression profile of the entire connexin family in order to identify the overall correlation tendency between connexin mRNA expression and tumour prognosis. In addition, we analysed the protein expression of Cx43, with particular emphasis on potential non-junctional functions due to, for example, nuclear localization. Our results highlight the fact that connexins show both pro- and anti-tumour propensities in lung cancer. This depends significantly on the connexin isoform and the lung cancer subtype. Moreover, protein expression and connexin localization are important from a biomarker perspective. Indeed, we show that Cx43 is localized to the nucleus in a subset of lung tumours, particularly in adenocarcinomas, and that this is associated with poor prognosis. Elucidation of the complex role of connexins in lung cancer is critical for the development of therapeutic approaches.

## 2. Results

### 2.1. Connexin mRNA Expression in Human Lung Tumours and Normal Healthy Tissue

We compared the mRNA expression levels of different connexins between healthy lung tissue and lung tumours with the online in silico analysis tool FireBrowseR (http://firebrowse.org/) using publically available The Cancer Genome Atlas (TCGA) data [53]. We observed significant changes in mRNA gene expression in a number of connexins (Table 1, and associated graphics in Supplementary Figure S1 that include expression variability and the presence of outliers). Our overall conclusions from these observations were verified using the TCGA Wanderer tool (http://maplab.imppc.org/ wanderer/) based on Illumina HiSeq RNA-seq analysis [54].

**Figure 1:**
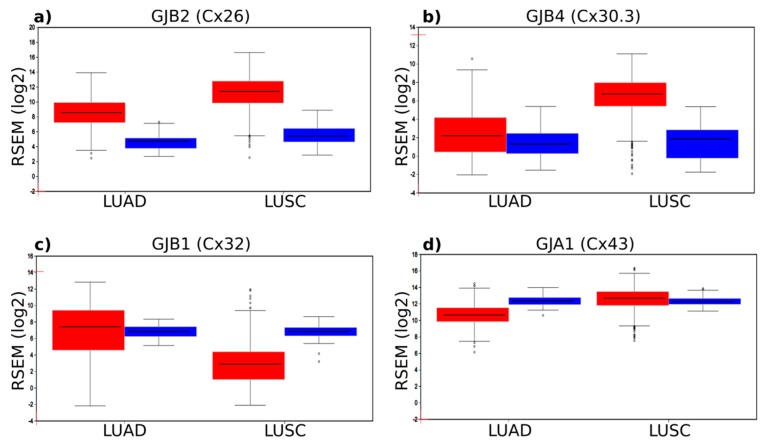
Connexin mRNA gene expression in normal tissue (blue) and in LUAD and LUSC tumours (red). (**a**) Expression of *GJB2* (encoding Cx26). Significant upregulation is seen in both tumour subtypes, particularly LUSC. (**b**) Expression of *GJB4* (encoding Cx30.3) is mainly upregulated in LUSC. (**c**) Expression of *GJB2* (encoding Cx32) is specifically downregulated in LUSC. (**d**) Expression of *GJA1* (encoding Cx43) is high in normal tissue but shows only a slight upregulation in LUAD and a slight downregulation in LUSC. The figure also highlights the occurrence of some outliers in tumours, such as very low expression of Cx26 and Cx30.3 in some LUSCs despite a general upregulation and very high expression of Cx32 in some LUSCs despite a general downregulation.

Compared with healthy tissue, a significant number of connexin genes were highly upregulated in the tumours, particularly in the LUSC subtype. A number of connexins displayed moderate downregulation, whereas other isoforms showed a mixed response dependent on the tumour subtype analysed. We focused on four connexin genes—Cx26, Cx30.3, Cx32 and Cx43—thought to play a role in lung cancer based on previous research (including mouse models). This group of four genes behaved very differently and clearly illustrated the varied changes in gene expression that can occur in connexin genes in lung cancer (Figure 1). Notably, compared with healthy tissue, there was a significant 14.4-fold and 63.5-fold upregulation of Cx26 mRNA expression in LUAD and LUSC, respectively (Figure 1a). GJB4 encoding Cx30.3 also displayed a significant 29.4-fold upregulation in LUSC (Figure 1b). This seemed to occur in several other beta-connexins (e.g., GJB6 encoding Cx30; see Table 1) that are typically expressed in differentiated squamous epithelial cells (e.g., skin keratinocytes). Cx26 and Cx30.3 have been suggested to be pro-tumorigenic in the lung, and the in silico data supported this notion based on the idea that overexpression may drive tumour progression. However, for GJB1 encoding Cx32, whose knockout mice are more susceptible to lung tumour formation, only a slight upregulation (1.45-fold) was seen in LUAD, whereas it was significantly downregulated in LUSC (0.064-fold) (Figure 1c). This further points to important connexin isoform and cancer subtype-specific differences. The role of GJA1 encoding Cx43 is controversial in lung cancer and, although this gene is highly expressed, there are only modest changes to its gene expression in the tumours (Figure 1d), with a slight downregulation in LUAD (0.31-fold) and slight upregulation in LUSC (1.33-fold). The overall changes in gene expression in all connexins is clearly diverse (Table 1), and it is likely that complex mechanisms, including epigenetic regulation, control the abundance of connexin mRNAs.

### 2.2. Regulation of Connexins at the DNA and mRNA Levels in Relation to Lung Cancer

**Figure 2:**
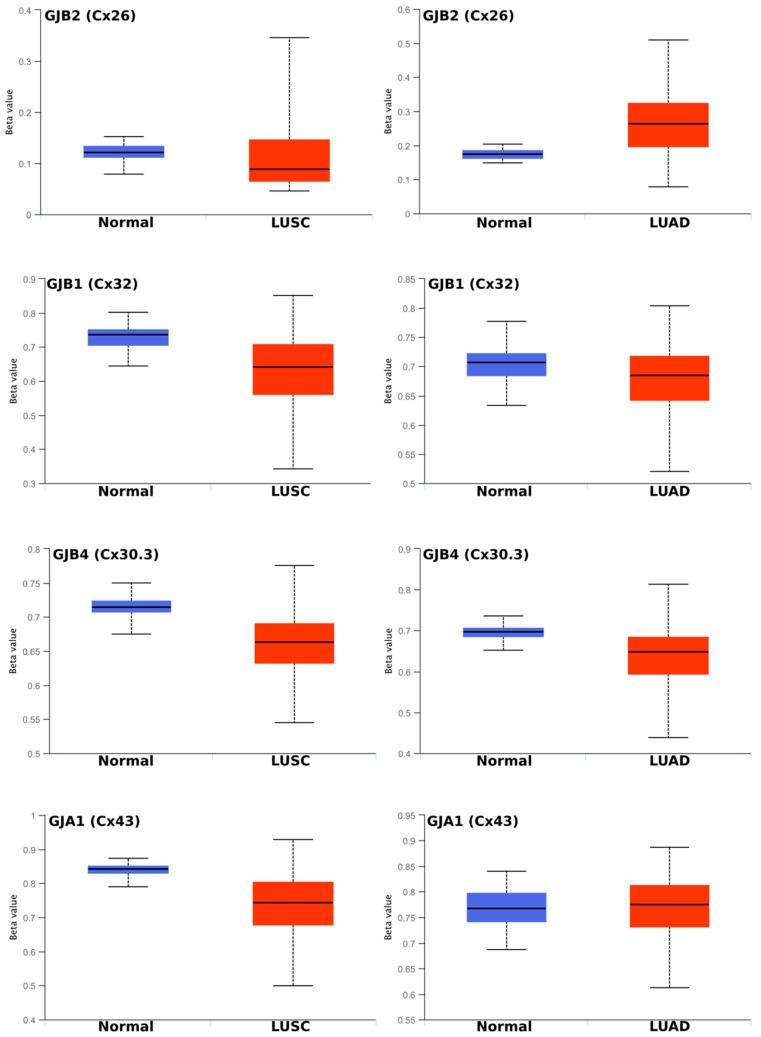
Methylation statuses of Cx26 (*GJB2*), Cx32 (*GJB1*), Cx30.3 (*GJB4*) and Cx43 (*GJA1*) in LUSC and LUAD compared with normal healthy lung tissue. The Beta value indicates the level of DNA methylation and ranges from 0 (unmethylated) to 1 (fully methylated). Different beta value cutoff values have been considered [56,57] to indicate hyper-methylation [Beta value: 0.5–0.7] or hypo-methylation [Beta value: 0.25–0.3].

We performed further in silico-based analysis of the four key lung cancer-associated connexins that we described in Section 2.1 and that showed significant changes in gene expression in healthy versus tumour tissues. One of the key regulators of gene transcription is gene methylation. We used an online (http://ualcan.path.uab.edu/) analysis tool [55] to gain insight into the correlation between the changes in connexin mRNA expression in lung tumours and changes in gene methylation. As seen in Figure 2, methylation-specific changes were connexin and tumour subtype-specific.

Moreover, no strict correlation could be inferred between methylation status and the observed gene expression or changes in gene expression (Figure 1). For example, *GJB2* (encoding Cx26) was dramatically upregulated in LUAD and especially in LUSC (Figure 1a). This partially correlated with a slight demethylation in LUSC (suggesting increased transcription). However, increased methylation (silencing) is seen in LUAD. Overall, however, *GJB2* seemed to be hypomethylated (a state considered [56,57] to occur with a Beta value in the range around or below 0.25–0.30), which favours trancription. *GJB1* (Cx32) appeared to be generally hypermethylated (a state considered [56,57] to occur with a Beta value in the range around or above 0.5–0.7). Some demethylation seemed to occur in LUSC (Figure 2b), but this actually contrasted with the loss of gene expression observed in this tumour subtype (Figure 1c). Better correlation was seen for *GJB4* (Cx30.3; Figure 2c) where some demethylation occurred (although the gene is still considered highly methylated), which corresponds to increased mRNA levels (Figure 1) in both tumour subtypes. *GJA1* (Cx43) is thought to be the most highly expressed connexin in lung tissue and tumours (Figure 1) yet it is highly methylated (Figure 2d).

We also used the TCGA Wanderer tool (http://maplab.imppc.org/wanderer/) [54] to analyse the methylation status of select connexins using individual probes. We confirmed our previous observation that the gene methylation status did not correlate well with the dramatic changes observed in gene expression (Supplementary Figure S2). For example, significant loss of Cx32 gene expression was observed in LUSC (see Table 1 and Figure 1c). However, all methylation probes—many of them with statistical significance, including those at the CpG island (green)—showed an overall reduced degree of methylation (more so in LUSC than in LUAD) (Supplementary Figure S2). This typically indicated increased rather than decreased transcription. Another example is Cx30, which showed a massive 289-fold upregulation in LUSC yet only minor changes in methylation status, and the most notable changes occured in LUAD rather than LUSC (Supplementary Figure S2). Other genes showed a better correlation. Notably, demethylation of GJB5 (particularly in LUSC) correlated with the observation that the mRNA expression of this gene was significantly upregulated in tumours (Supplementary Figure S2).

Small individual regions can be sufficient to significantly influence transcription. Indeed, although there were no major overall changes in the methylation status of GJB2 (encoding Cx26; Figure 2a), a noticeable demethylation was seen in probe cg24425972 in LUSC (Figure 2a,b) compared with normal tissues, which correlates well with the observed significant upregulation of the mRNA expression-based Illumina HiSeq RNA-seq (Figure 2b, see also Figure 1a). A similar pattern was seen in LUAD where there was slightly less demethylation, which corresponds to the less significant upregulation of mRNA in this tumour type (Supplementary Figure S2).

We also screened whether any connexin gene has been identified to show any correlation between methylation and clinical features using FirebrowseR (http://firebrowse.org/) [53]. Indeed, among only 30 genes identified, methylation of GJB2 (encoding Cx26) was associated with tumour stage in LUAD (Kruskal-Wallis *p*-value = 0.0001923, *Q* value = 0.119). Thus, although there was a poor direct correlation between mRNA expression and methylation status in many connexin genes, specific correlations could be identified and may be biologically and clinically significant.

### 2.3. Connexin mRNA Expression Is Associated with Both Poor and Good Prognosis, Which Depends on Both Lung Cancer Subtype and Connexin Isoform

We used a dedicated lung cancer-specific Kaplan-Meier survival analysis tool [58] to correlate connexin mRNA expression (from arrays, *n* = 2435) with the OS of patients. We analysed all gap junction genes with valid probes available (*n* = 18). The overall results for lung cancer as a whole are summarised in Table 2. A substantial amount of insightful information can be drawn from this summary. First, there are major differences between different connexin isoforms. Some connexins are associated with better prognosis, whereas others are associated with poor prognosis. Critically, this seems highly dependent on the tumour subtype.

**Figure 3:**
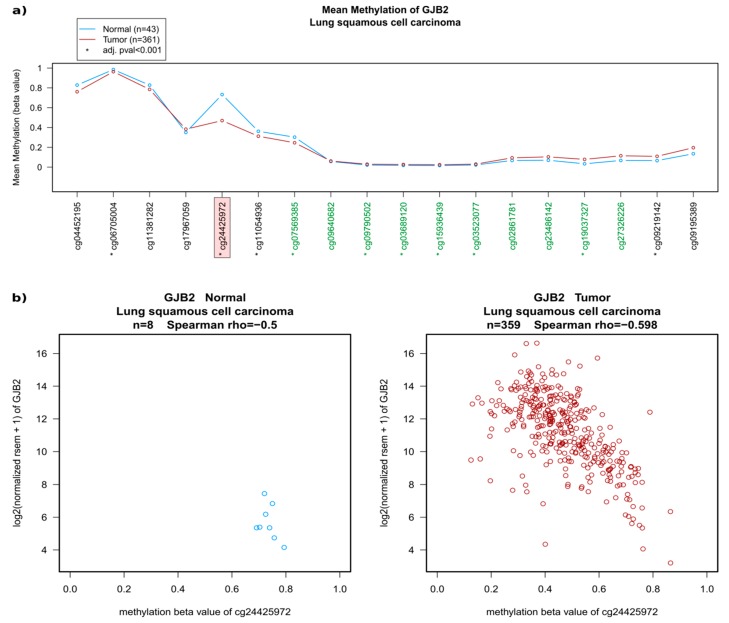
Example of the methylation status of Cx26 (*GJB2*) in LUSC. (**a**) A 450k Methylation Array displaying individual probes along the gene region is shown (equally distributed). Probes in green are part of the CpG cluster of this gene. Significant differences are highlighted with an asterisk on individual probes. Probe cg24425972 (highlighted by the pink box) shows the most significant demethylation in LUSC compared with healthy tissue. (**b**) The methylation status of probe cg24425972 is clearly reduced in most tumour samples (red, right side) compared with healthy tissue (blue, on the left) and this correlates well with the observed increased gene expression in the samples that have reduced methylation (RSEM from Illumina HiSeq RNA-seq).

We paid particular attention to connexin genes known to play a role in lung cancer, namely, Cx26, Cx30.3, Cx32 and Cx43. Of these, only Cx43 was associated with better prognosis in both the LUSC and LUAD subtypes (Figure 3a). However, the relatively small change in the hazard ratio (HR, chance of death) suggests that this association has quite a low impact (although it is highly significant statistically).

This correlates with the rather minimal change in gene expression between healthy and tumour tissue (Figure 1d). On the other hand, high Cx32 expression predicted better survival in overal NSCLC and LUAD patients, whereas LUSC patients had a tendency to perform worse if grouped in the cohort with high Cx32 expression (Figure 3b). The recent findings that Cx30.3 is linked to tumour progression [46] are strongly supported by our in silico analysis, which predicted significantly worse survival in the NSCLC group and the LUAD subtype (Figure 3c). Notably, other connexins (such as Cx31, known to be expressed in lung cancer) seemed to follow a similar pattern (Table 2). Cx26 predicts a poor outcome for NSCLC in general, and a significantly worse prognosis in the LUAD subgroup (HR = 2.12), where patients with high Cx26 expression are predicted to live an average of 56 months less than those with low Cx26 expression (80 months vs. 136 months). Of note, similar to Cx30.3, Cx26 seemed to be significantly overexpressed in tumour tissue versus healthy tissue (Figure 1). Cx26 was significantly more upregulated in LUSC versus healthy tissue (63.5-fold), yet this was not asssociated with a statistically significant effect on the HR (if anything, the high Cx26-expressing cohort showed a tendency for increased survival). All other connexins analysed are represented in Supplementary Figure S3. These observations were supported by data from The Pathology Atlas of the Human Protein Atlas database (https://www.proteinatlas.org/pathology). This open-access database (retrieved from TCGA) contains correlation analyses based on mRNA expression levels with respect to clinical outcome for 17 major cancer types and almost 8000 cancer patients [59]. The database identifies GJB2 as significantly associated with poor prognosis in lung cancer (*p* = 6.25 × 10^−4^). This remained statistically significant in LUAD (*p* = 1.45 × 10^−5^) but not LUSC after subclassification. Of interest, of all the other connexin genes, this database also identified GJB3 (Cx31) to be significantly associated with poor prognosis in lung cancer (*p* = 4.56 × 10^−4^) and in LUAD (*p* = 3.54 × 10^−8^) but not in LUSC. This corresponds well with our own analysis (Table 2). Overall, these results clearly suggest that connexins that are upregulated in the tumours may affect (or at least associate with) the clinical outcome very differently in distinct tumour subtypes. Additionally, it is clear that the association between connexin expression and prognosis was much more clear-cut in LUAD than in LUSC (Figure 3, Table 2 and Supplementary Figure S3).

Finally, in order to further corroborate the array-based analysis, we analysed RNA-seq data from a Pan-cancer data collection available online (http://kmplot.com). We specifically focused on lung cancer (LUSC, *n* = 501; LUAD, *n* = 513). For some genes, we observed significant discordance between the gene expression and the HR in the two datasets, including for genes that had a high HR with a highly significant *p*-value in the array (Supplementary Table S1). Notably, for Cx43, the array analysis indicated an association with better prognosis in LUAD (HR = 0.64, highly significant) yet, in the RNA-seq analysis, this showed a tendency for poor prognosis (HR = 1.34), although this was not deemed significant (*p* = 0.07). However, for the three other connexin genes we have focused on (GJB1, GJB2 and GJB4) showed clear concordance.

### 2.4. Connexin Protein Expression and Subcellular Localization

**Figure 4:**
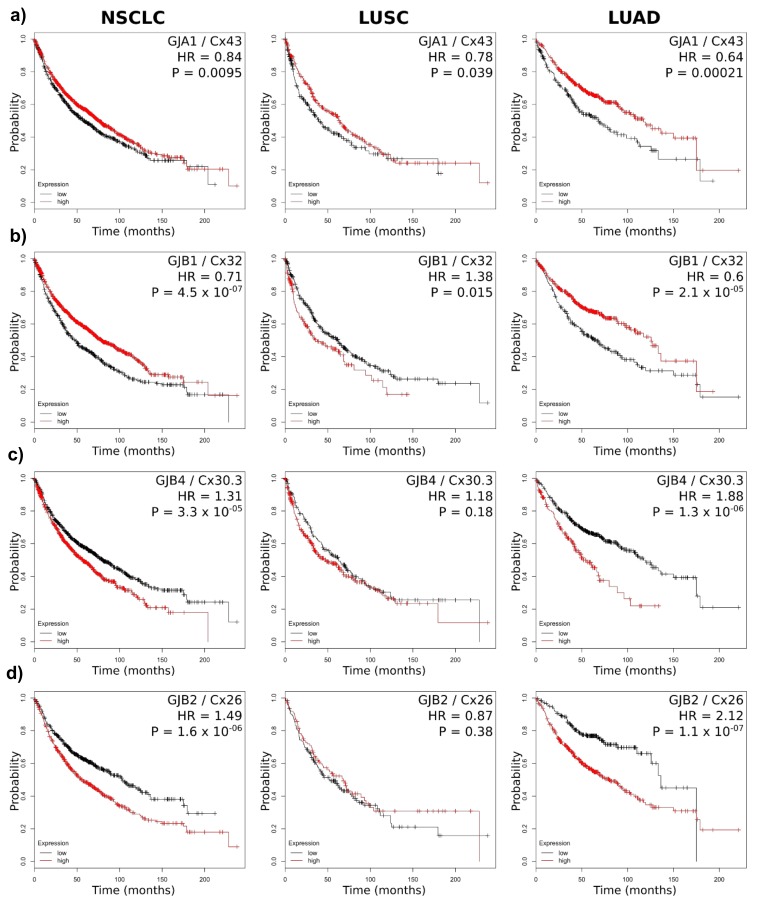
Kaplan-Meier curves of connexins known to drive lung tumorigenesis. The cohort expressing high levels of connexin mRNA is depicted in red, and the cohort with low connexin levels is in black. Probability refers to the likelihood of being alive at any time point. A hazard ratio (HR) below 1 suggests an association with better prognosis, whereas a HR above 1 suggests better prognosis (in the cohort expressing high levels of connexins). (**a**) Cx43; (**b**) Cx32; (**c**) Cx30.3; and (**d**) Cx26.

Cx43 is implicated in lung cancer but substantial evidence also indicates that Cx43, in a highly context-dependent manner, can act as both a tumour supressor and tumour promoter [16]. Our mRNA array analysis suggested that Cx43 is weakly associated with better prognosis, especially in LUAD (Figure 4a). However, our RNA-seq analysis did not support these findings and suggested a potential association with poor prognosis in LUAD (Supplementary Table S1). Ultimately, analysis of the protein expression of Cx43 is needed to provide better insight into the role of Cx43 and its correlation with cancer. Cx43 is the most widely expressed connexin in lung epithelia, and several studies indicate dysregulation at multiple levels: transcription (including methylation), translation (including internal translation of truncated isoforms) and post-translational modifications (notably phosphorylation) [4]. These alterations may affect Cx43 expression levels, function and subcellular localization. In order to gain further insight into the underlying features of Cx43 in cancer, which may explain the rather weak association at the mRNA level, we also performed an in depth analysis of Cx43 at the protein level. We performed an immunohistochemistry (IHC) study of Cx43 on a tissue microarray consisting of 73 tumours (50 LUADs and 23 LUSCs). We used a validated Cx43 antibody that was absent in paraffin-embedded cells negative for Cx43. This antibody targets the last 20 amino acids of human Cx43, has been extensively used in other IHC studies (e.g., [48,60,61]) and was found in the Protein Atlas (https://www.proteinatlas.org/) to show an excellent correlation between mRNA expression and protein expression [59].

**Figure 5:**
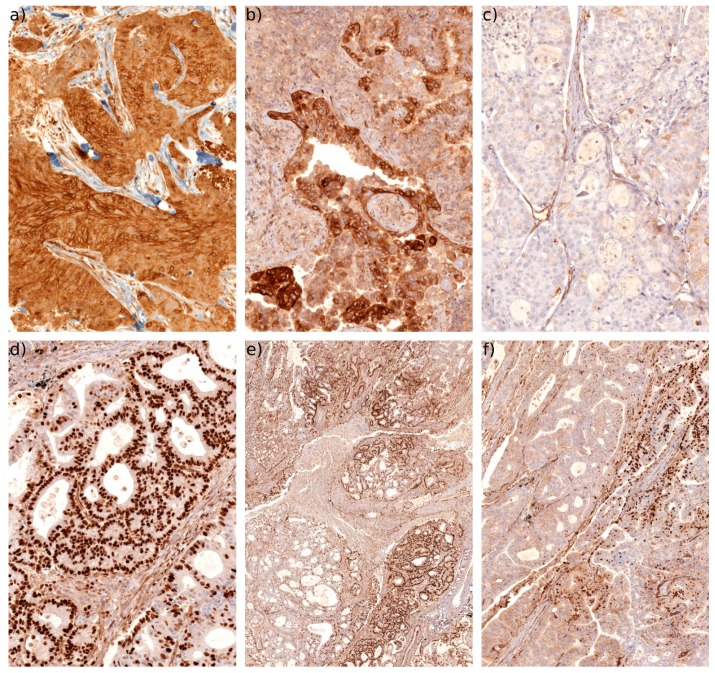
IHC of Cx43. (**a**) Typical example in LUSC demonstrating intense Cx43 staining with a predominantly membranous and cytoplamsic pattern (20×). (**b**) Typical example in LUAD of a patchy high Cx43 expression with a predominantly cytoplasmic expression pattern (20×). (**c**) Example of a LUAD with very low or negative Cx43 expression (20×). Note that stromal cells such as endothelial cells are still positively stained. (**d**) Typical example of high levels of nuclear Cx43 expression in LUAD (20×). Note that some non-tumour cells are also sometimes positive for nuclear Cx43. (**e**) Low magnification overview (4×) of a LUAD with significant areas of nuclear Cx43 expression. (**f**) Area of the same tumour (10×) where nuclear Cx43 can be observed together with areas either negative for Cx43 or with low cytoplasmic levels.

The array consisted of triplicate punch biopsies of each tumour covering the core of the tumour. The Cx43 expression intensity varied widely (and was scored as 0: absent, 1: weak, 2: medium, and 3: intense staining). Some tumours expressed significant levels of Cx43 with a typical pattern in the membrane and cytoplasm (Figure 5a). Some tumours showed areas of very high expression (Figure 5b) typically concentrated in the cytoplasm. Other tumours expressed very low or absent levels of Cx43 (Figure 5c). Stromal cells (e.g., immune cells, muscle cells and endothelial cells) were also frequently shown to express Cx43, as expected (Figure 5c). This is consistent with previous reports in the lung [34,37]. Our analysis suggested that the expression levels per se did not significantly correlate with the tumour grade or OS. This may be due to the low number of cases. However, we noted a significant number of tumours (5 of the 23 LUSCs and 21 of the 50 LUADs) with a highly pronounced pattern of nuclear Cx43 expression (Figure 5d). This protein could be expressed throughout most of the tumour (Figure 5e) or in more isolated areas (quantified as positive if more than 5% of the tumour expressed nuclear Cx43). Some tumours diplayed areas of nuclear Cx43, areas of cytoplasmic Cx43 and areas negative for Cx43 (Figure 5f). We could not correlate the presence of nuclear Cx43 with a specific tumour subtype or stage (i.e., we detected nuclear Cx43 in both low grade and high grade tumours, as well as in various LUAD subtypes such as acinar, papillary, micropapillary and solid). Moreover, other stromal cells (e.g., immune cells, endothelial cells and chondrocytes) also occasionally expressed nuclear Cx43 (Figure 5), suggesting that this nuclear translocation is driven by microenvironmental cues.

**Figure 6:**
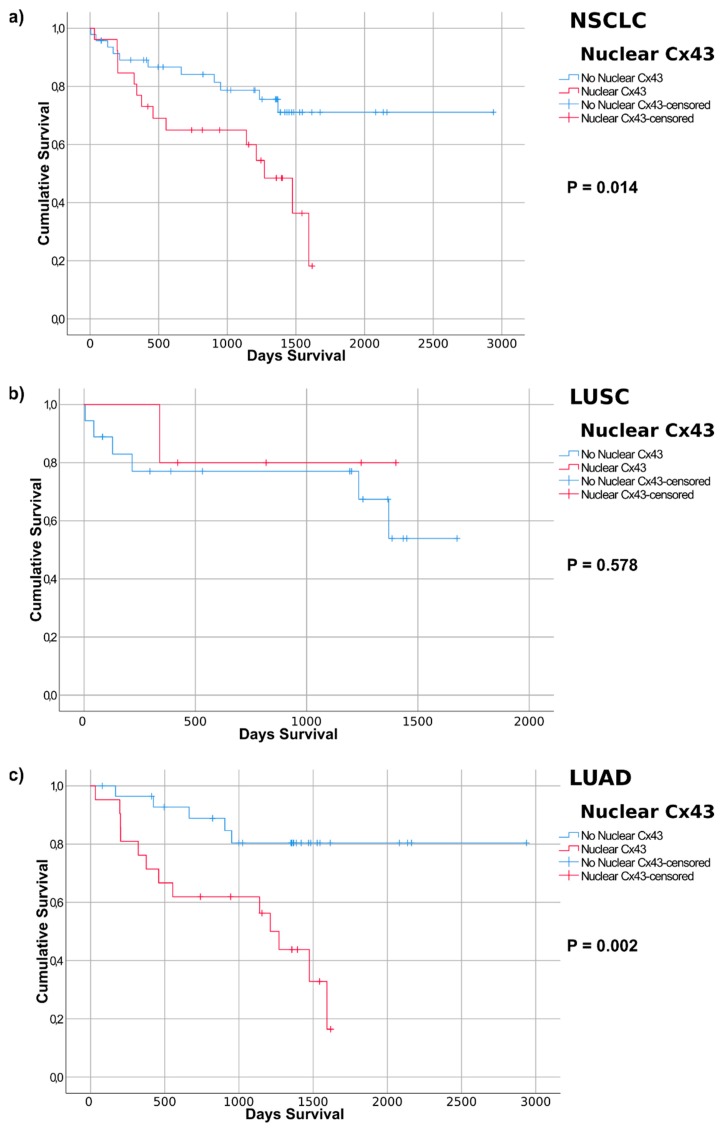
Kaplan-Meier curves based on nuclear Cx43 expression detected by immunohistochemistry of lung cancer samples. Tumours with significant nuclear Cx43 expression (over 5% of total tumours) are depicted in red and compared with the rest of the tumour samples (in blue). *p*-values refer to the log-rank test (Mantel-Cox analysis). Average survival times of the patient cohorts are listed. (**a**) NSCLC (non-small cell lung cancer, refers to the merged LUAD and LUSC groups); (**b**) LUSC (lung squamous cell carcinoma); (**c**) LUAD (lung adenocarcinoma).

We performed bivariate correlation analysis comparing basic tumour and patient features and using different Cx43 staining parameters (Supplementary Table S2). Notably the expression of Cx43 (in the cytoplasm/membrane (CM) alone, or in combination with nuclear Cx43) is statistically higher in LUSC compared to LUAD. The presence of CM Cx43 immunoreactivity also correlates with a higher Ki67 score (suggesting Cx43 is expressed at higher levels in highly proliferating tumours). This is not significant for nuclear Cx43 alone or in combination with CM Cx43 staining. A larger cohort and additonal clinicopathological markers may indicate additional correlations in this aspect. Our overall aim was to compare IHC with OS, compared to OS obtained from the in silico analysis. For this we used Kaplan Meier survival curves to look for associations between the Cx43 staining pattern and OS. We identified a strong association between nuclear Cx43 and OS. Our Kaplan-Meier survival curve analysis (Figure 6a) indicated that nuclear Cx43 was significantly associated with poor OS in NSCLC (*p* = 0.014).

We then analysed OS after stratification into LUAD and LUSC tumour subtypes. No association was found between nuclear Cx43 and poor OS in LUSC (*p* = 0.578). In LUAD, however, nuclear Cx43 was significantly associated with poor survival (*p* = 0.002). There was no significant correlation between CM Cx43 intensity and OS in NSCLC, LUSC or LUAD (based on using either moderate and high, or mild, moderate and high, Cx43 intensity, Supplementary Figure S4a–f), although the trend suggested Cx43 was associated with poor survival. When including patients that scored positive for either CM Cx43 IHC or nuclear Cx43, no significance was observed in NSCLC, LUSC or LUAD (Supplementary Figure S4g–i). However, patients classified into a single group positive for either nuclear Cx43 staining or moderate to strong CM staining, showed significant association for poor prognosis in NSCLC (*p* = 0.008) and LUAD (*p* = 0.005) (Supplementary Figure S4j–l). Again LUSC was not significant, which may be due to the few patients in this cohort. This suggests Cx43 intensity may also be a factor related to outcome.

## 3. Discussion

This study analysed connexins to identify specific changes in relation to lung cancer at the DNA (methylation), RNA (expression) and protein (expression and subcellular localization) levels. Several important observations have been made. It seems clear that the mRNA expression of a number of connexins are dramatically changed in tumours (up to 289-fold in the case of *GJB6*/Cx30 in LUAD). These changes vary significantly according to tumour subtype (LUAD versus LUSC). Considering the important role of connexins in tissue homeostasis and cancer, these observations merit further investigation, including functional studies. It will be important to determine whether this is a mere association or whether causation is implicated. Regardless, these changes suggest that a number of connexins, particularly those showing a significant upregulation in tumours compared with healthy tissue, may serve as potentially useful prognostic or diagnostic biomarkers.

Mechanistically, the regulation of the changes in connexin mRNA expression in tumour versus healthy tissue, or indeed between tumour subtypes, remains poorly understood. We investigated the role of connexin promoter methylation due to its well-known and important role as a regulator of gene expression. Surprisingly, the correlation between connexin gene expression and connexin gene methylation was poor. For instance, highly expressed connexin genes were found to be hypermethylated while genes with low expression were hypomethylated. This is the opposite of what would be expected. Moreover, the changes in methylation between healthy and cancer tissues did not correlate with the corresponding changes in gene expression. For instance, significant upregulation of a connexin did not tend to correlate with demethylation. A certain correlation could be seen in some genes (e.g., *GJB2* encoding Cx26), particularly when looking at specific probes. However, functional studies are required to validate the significance of these observations. The direct correlation between methylation status and transcription in cancer seems to be restricted to a subset of genes in a tissue-specific manner, and the exact underlying mechanism remains elusive [62]. This seems to be true for connexins as well, and further connexin gene-specific studies are required to determine any clinical value.

Functionally, little is known regarding the role of most connexins in relation to lung cancer. We addressed their role from an association standpoint only. However, the clear associations observed in many cases suggest that connexins may be causally implicated in lung cancer. Indeed, this is supported by some functional studies. Notably, results from lung cancer mouse models correlate well with the prognostic prediction for Cx32 and Cx43 made at the mRNA level [16,26,27,29,30,31,63]. However, some connexins significantly upregulate their mRNA expression in lung tumours and are associated with poor prognosis. The pathophysiological correlation of this association is unclear. By correlating connexin mRNA expression with prognosis, we highlight connexins (e.g., Cx31) that may need to be studied further, including at the protein level by IHC. However, it is important to keep in mind that the correlations currently described are mere associations that do not imply causation. Moreover, mRNA expression is typically derived from all cells present in the tumour sample (e.g., in the lung, there would be a mix of pneumocytes, bronchial epithelium, endothelial cells, macrophages and other cell types). Indeed, a recent study showed that cancer-associated fibroblasts can form Cx43-mediated gap junctions with NSCLC cells to support their malignant progression [64]. The ratios of these cell types in a tumour change. Going forward, protein expression (IHC) and specific functional studies (in vitro and in vivo models) are critical elements that need to be addressed.

Our proof-of-principle study, looking at Cx43 protein expression in lung tumours, made a highly surprising and important discovery: a statistically significant association between nuclear Cx43 expression and reduced OS. The very distinct and clear nuclear Cx43 expression profile, present only in some patients, makes it an excellent putative biomarker for the disease. However, more extensive studies are needed to verify its use in lung cancer (and potentially other cancer types). Notably, the sample size needs to be increased, and further sub-characterization of tumour histology and other clinicopathologic parameters needs to be correlated with Cx43 expression. It is also noteworthy that we observed nuclear Cx43 in non-tumoural cells (e.g., endothelial cells, chondrocytes and immune cells) in those tumours displaying nuclear Cx43. This suggests that there may be microenvironmental conditions that regulate the nuclear translocation of Cx43. Indeed, in glioma tumours, nuclear Cx43 was observed in areas where leukocytes were present [47].

Our IHC study also showed the importance of not relying on only the mRNA expression profile. Indeed, the array analysis suggested (with high statistical significance) that high levels of Cx43 are associated with better prognosis. Clearly, this does not take into consideration protein localization and subpopulation of patients, with Cx43 protein localized to the nucleus seemingly associated with poor prognosis. Moreover, and unlike IHC studies that specifically score the tumour cells, most studies at the RNA level tend to average the entire tissue section, leading to significant cross-contamination by other cells (e.g., endothelial and immune cells, known to express Cx43 and to be altered in a tumour environment). The reliability of array probes also needs to be carefully considered. Indeed, our Kaplan-Meier survival analysis using RNA-seq data suggested that Cx43 may be associated with poor prognosis in LUAD (in contrast to the array analysis, but in concordance with our nuclear Cx43 association study). The reason for this discrepancy for Cx43 in LUAD is not clear. As seen here, both approaches can give insightful information, but both will require substantial follow-up before any clinical use.

A number of studies have described nuclear Cx43 in cell lines [50], including lung cancer cells [51]. Nuclear Cx43 has additionally been detected in some tumours, notably in glioma [47] and colon cancers [48], although these studies did not report an association with overall survival. Truncated forms of Cx43 have also been detected in the nucleus of cancer cells [52]. This is particularly interesting in light of recent reports demonstrating that the *GJA1* transcript that encodes for Cx43 also independently encodes for truncated isoforms [6,7,8]. We showed that this also occurred in lung cancer cells and could be regulated by modulating specific cancer signalling pathways such as mTOR and MAPK-MNK1/2 [7]. Moreover, the major truncated Cx43 isoform, GJA1-20k, was recently experimentally shown to translocate to the nucleus and act as a direct transcriptional activator of N-cadherin in vivo [13]. N-cadherin is associated with EMT in cancer [65], predicts poor prognosis in lung cancer [66] and may be a therapeutic target in chemoresistant NSCLC [67]. Indeed, our Kaplan-Meier analysis of TCGA array data associated N-cadherin with poor prognosis in lung cancer (data not shown). It will be of considerable interest to test whether nuclear Cx43 is correlated with N-cadherin expression in lung tumours. Because the antibody used for IHC detects the C-terminus of Cx43, we cannot directly determine whether the nuclear signal is due to the presence of full-length Cx43 or any truncated form such as GJA1-20k. Further studies are needed to address this question. Nevertheless, the link between nuclear Cx43 expression and prognosis opens up putative therapeutic options aimed at restoring Cx43 trafficking to the membrane. This approach is particularly tantalizing because it may serve both to disrupt the nuclear signalling cascade associated with poor prognosis and to restore GJIC and tissue homeostasis. We have identified several FDA-approved drugs that affect the translation of truncated Cx43 isoforms and future studies will address whether they can modify nuclear Cx43 localization or the tumour phenotype.

## 4. Materials and Methods

### 4.1. Immunohistochemistry

IHC was performed using the avidin-biotin-peroxidase technique. Five-micron-thick sections were cut from formalin-fixed, paraffin-embedded cell pellets and mounted on poly-L-lysine-coated glass slides. Sections were deparaffinized in xylene and rehydrated in graded alcohol. Endogenous peroxidase was blocked by immersing the sections in 0.1% hydrogen peroxidase in absolute methanol for 20 min. For antigen retrieval, the tissue sections were heated in a pressure cooker in 10 mM citric acid monohydrate, pH 6.0, for 5 min and then incubated with primary antibodies for 60 min at room temperature. IHC was performed with the Benchmark XT slide stainer (Ventana Medical Systems, Inc., Tucson, AZ, USA). The primary antibody used was anti-Cx43 (C6219, Sigma-Aldrich Quimica SL, Madrid, Spain) at 1:1000 dilution. All slides were haematoxylin-counterstained, dehydrated and mounted. Negative controls were performed by omitting the primary antibody and showed minimal non-specific signal. The immunostaining intensity was scored as follows: negative, 0; weak, 1; moderate, 2; and intense, 3. Nuclear Cx43 expression was considered to be significant when more than 5% of the tumour samples showed nuclear staining.

### 4.2. Statistics

Statistical studies were performed with the Statistical Package for the Social Sciences (SPSS 25.0; SPSS Inc., Chicago, IL, USA). Statistical significance for the Kaplan-Meier curves were considered at *p* < 0.05 using log-rank (Mantel-Cox) analysis. Bivariate correlations were analysed using two-tailed Pearson’s correlation coefficient with significance set at *p* < 0.05.

### 4.3. Human Tissue Specimens and Tissue Microarray

Tissue microarrays containing 73 cases of primary lung cancer were obtained from the pathology department. Triplicates of each tumour were used in the tissue microarray. All of the tissue specimens for this study were obtained with informed patient consent from the Hospital Vall d’Hebron Biobank, and the use of these specimens for this study was approved by the ethics committee of Hospital Vall d’Hebron (PR(AG)327/2014).

### 4.4. Bioinformatic Analysis

Gene expression analysis: Connexin mRNA expression in lung tumours and in corresponding normal tissues was analysed by using data from The Cancer Genome Atlas (TCGA) using FireBrowseR (http://firebrowse.org/) following the standard input and output values defined by the software [53]. Our observations were verified using the TCGA Wanderer tool (http://maplab.imppc.org/wanderer/) based on Illumina HiSeq RNA-seq analysis [54].

Kaplan-Meier survival curves (mRNA expression): Overall survival (OS) was derived both from TCGA array data of lung cancer and from pan-cancer RNA-seq data. All analyses were performed online (http://kmplot.com/) using the standard setting as defined by the software [58], with the following exception: Patients were split using “Auto select best cutoff”. Survival refers to OS. The cutoff for significance was set to a strict *p* < 0.001. Additional verifications and analyses were performed using the Pathology Atlas of the Human Protein Atlas database (https://www.proteinatlas.org/pathology) using a standard setting and a strict cutoff value for significance set to *p* < 0.001. This open-access database (retrieved from The Cancer Genome Atlas [TCGA]) contains correlation analyses based on mRNA expression levels with respect to clinical outcome for 17 major cancer types and almost 8000 cancer patients [59].

Methylation analysis: We used an online (http://ualcan.path.uab.edu/) analysis tool [55] to gain insight into the correlation between changes in connexin mRNA expression in lung tumours and changes in gene methylation. Standard pre-determined settings were used. We used the TCGA Wanderer tool (http://maplab.imppc.org/wanderer/) [54] to analyse the methylation status of individual probes and the correlation to gene expression in the tumours.

## 5. Conclusions

In conclusion we have found that major changes in some specific connexin mRNAs often occur in lung tumours but in general this do not correlate well in relation to changes in promoter methylation. Connexin mRNA expression can however correlate with both good and poor prognosis, which depends on the connexin isoform analysed and the histological subtype (LUAD versus LUSC). In addition to changes in mRNA expression, it is clear that protein location and functionality is critical. In this study, we identified a subset of tumours that exhibited a unique strong nuclear Cx43 expression pattern that predicted worse overall survival. The prognostic value was highly significant in LUAD, and larger cohorts will be needed to definitively assess the correlation in LUSC. This study highlights the importance of analysis of connexin expression at the protein level, particularly the subcellular localization. It also proposes that modulation of Cx43 trafficking may be a useful therapeutic strategy.

## Figures and Tables

**Table 1:** Summary of fold-changes in connexin mRNA expression in lung adenocarcinoma (LUAD) and lung squamous cell carcinoma (LUSC) compared with normal healthy tissue.

Regulation	Cx	Gene	LUAD	LUSC
Up	Cx46	*GJA3*	1.74	35.6
	Cx59	*GJA9*	3.01	1.82
	Cx26	*GJB2*	14.4	63.5
	Cx31	*GJB3*	3.27	34.4
	Cx30.3	*GJB4*	1.88	29.4
	Cx31.1	*GJB5*	1.66	59.9
	Cx30	*GJB6*	7.47	289
Down	Cx37	*GJA4*	0.36	0.18
	Cx40	*GJA5*	0.32	0.17
	Cx50	*GJA8*	0.83	0.97
	Cx45	*GJC1*	0.67	0.76
	Cx47	*GJC2*	0.325	0.234
	Cx30.2	*GJC3*	0.817	0.61
	Cx31.9	*GJD3*	0.52	0.43
	Cx40.1	*GJD4*	0.89	0.96
Mixed	Cx43	*GJA1*	0.31	1.33
	Cx32	*GJB1*	1.49	0.064
	Cx25	*GJB7*	0.41	7.18
	Cx36	*GJD2*	0.56	2.9

**Table 2:** Summary of the overall survival (OS) of patient cohorts grouped into cohorts expressing either high or low levels of connexin mRNA in non-small cell lung cancer (NSCLC) in general, lung adenocarcinoma (LUAD) or lung squamous cell carcinoma (LUSC) compared with normal healthy tissue.

Gene	Connexin	HR NSCLC	OS Change (Months) High Cx	HR LUSC	OS Change (Months) High Cx	HR LUAD	OS Change (Months) High Cx
*GJA1*	Cx43	0.84 *	+19	0.78	+24	0.64 *	+49
*GJA3*	Cx46	1.42 *	−41	0.77	+27	1.48 *	−42
*GJA4*	Cx37	0.67 *	+43	0.83	+22	0.67 *	+45
*GJA5*	Cx40	1.30 *	−23	1.20	−4	1.86 *	−69
*GJA8*	Cx50	1.18	−11	1.16	−17	0.60 *	+59
*GJA10*	Cx62	1.20	−28	0.77	+24	1.62 *	−52
*GJB1*	Cx32	0.71 *	+31	1.38	−24	0.60 *	+59
*GJB3*	Cx31	1.56 *	−37	1.27	−21	2.39 *	−114
*GJB4*	Cx30.3	1.31 *	−23	1.18	−15	1.88 *	−62
*GJB5*	Cx31.1	1.52 *	−36	1.12	−12	1.63 *	−57
*GJB6*	Cx30	1.40 *	−38	0.75	+29	0.82	+11
*GJB2*	Cx26	1.49 *	−47	0.87	+16	2.12 *	−56
*GJC1*	Cx45	0.73 *	+36	1.22	−15	0.66 *	+37
*GJC2*	Cx47	1.13	−6	0.81	+27	1.45 *	−41
*GJC3*	Cx30.2	1.26 *	−27	0.78	+8	1.76 *	−53
*GJD2*	Cx36	1.25 *	−18	1.14	−11	2.13 *	−68
*GJD3*	Cx31.9	0.68 *	+39	0.66 *	+24	1.26	−44
*GJD4*	Cx40.1	1.25 *	−22	0.83	+27	1.49 *	−33

1.Abbreviations: HR, hazard ratio; OS, overall survival; NSCLC, non-small cell lung cancer; LUSC, squamous cell lung cancer; LUAD, adenocarcinoma lung cancer; Cx, connexin. 2. The table depicts the patient hazard ratio, where the cohort expressing high levels of connexin mRNA is either associated with better survival (depicted in green, HR less than 1, i.e., less chance of death, suggesting the connexin is a tumour suppressor) or with worse survival (red, HR greater than 1, i.e., higher chance of death, suggesting the connexin is pro-tumorigenic). The average change in survival (months) is the difference between the patient cohorts grouped as having high and low levels of connexins. Those associations with a significant statistical difference (*p* < 0.01) are in bold and marked with an asterisk *. Further detail and individual Kaplan-Meier curves can be seen in Supplementary Figure S3.

## Supplementary Materials

The following are available online at https://www.mdpi.com/2072-6694/11/3/320/s1, Figure S1: Connexin mRNA expression in healthy lung tissue and corresponding lung tumour subtypes LUAD and LUSC, Figure S2: Examples of methylation statuses of key connexin genes in healthy versus tumour tissue, Figure S3: Kaplan-Meier curves of connexins known to drive lung tumorigenesis, Figure S4: Kaplan-Meier curves based on Cx43 expression patterns detected by immunohistochemistry of lung cancer samples, Table S1: Correlation between HR survival curves between Microarray based versus RNA-seq based analysis, Table S2: Pearson Correlation.
