# Supplementary material for: Insight into the Role and Regulation of Gap Junction Genes in Lung Cancer and Identification of Nuclear Cx43 as a Putative Biomarker of Poor Prognosis
Source: Cancers (Basel). 2019 Mar 6;11(3):320. doi: 10.3390/cancers11030320 (PMC6468764; doi:10.3390/cancers11030320)
Supplement: Supplementary file 1 [file cancers-11-00320-s001.zip › cancers-453559-Supplementary Figures.pdf]

cancers MDPI

Supplementary Materials: Insight into the Role and Regulation of Gap Junction Genes in Lung Cancer and Identification of Nuclear Cx43 as a Putative Biomarker of Poor Prognosis

Trond Aasen, Irene Sansano, Maria Ángeles Montero, Cleofé Romagosa, Jordi Temprana-Salvador, Alexandre Martínez-Marti, Teresa Moliné, Javier Hernández-Losa and Santiago Ramón y Cajal

Supplementary Figure S1.

Connexin mRNA expression in healthy lung tissue (blue) and corresponding lung tumour subtypes LUAD and LUSC (red). The figure corresponds to Table 1 (and are grouped into (**A**) increased, (**B**) deacreased or (**C**) mixed changes in connexin expression in LUSC and LUAD compared to healthy tissue).

A) Increased expression in LUAD and LUSC tumours:

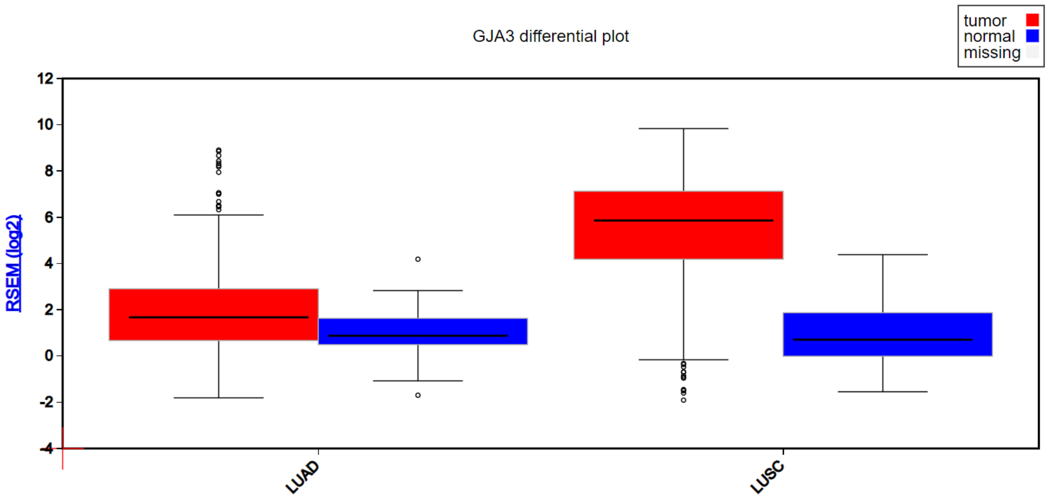

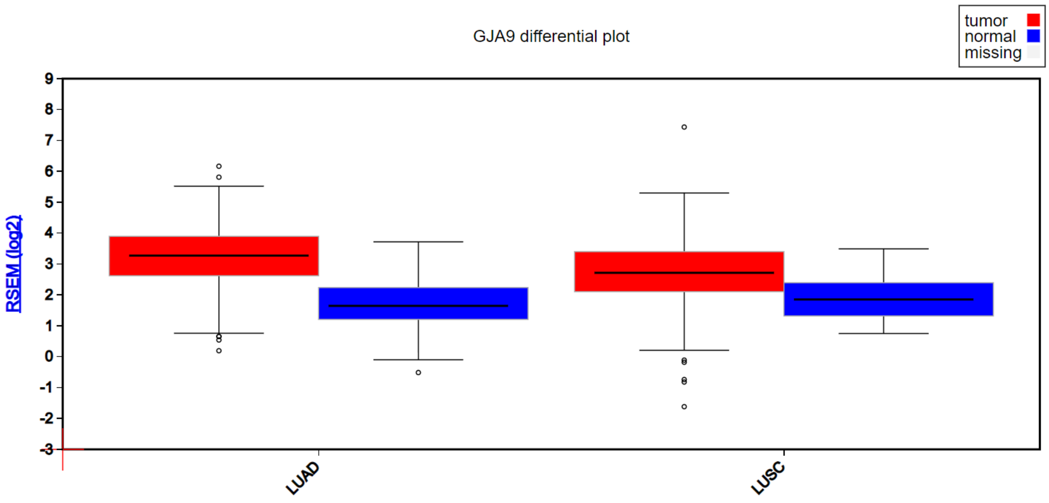

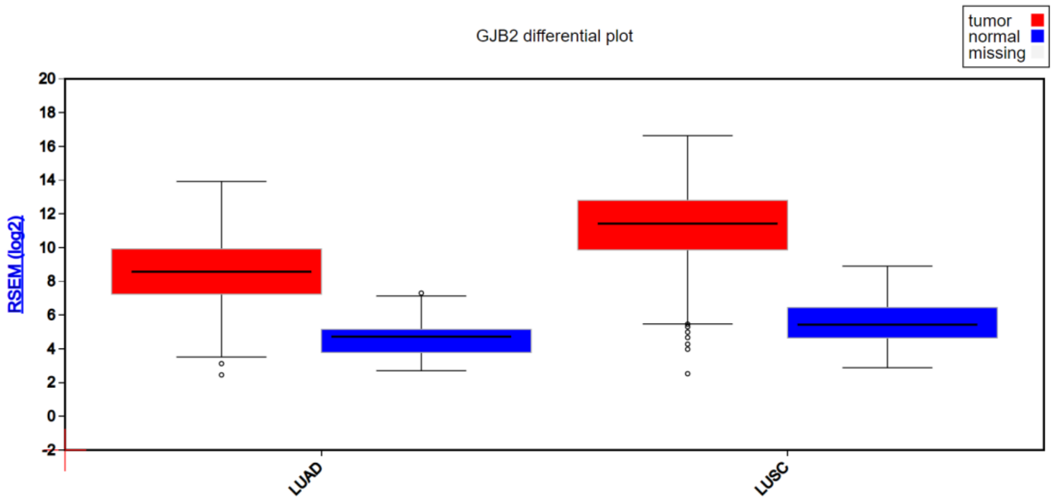
GJB2 differential plot
tumor
normal
missing
RSEM (log2)
20
18
16
14
12
10
8
6
4
2
0
-2
LUAD
LUSC

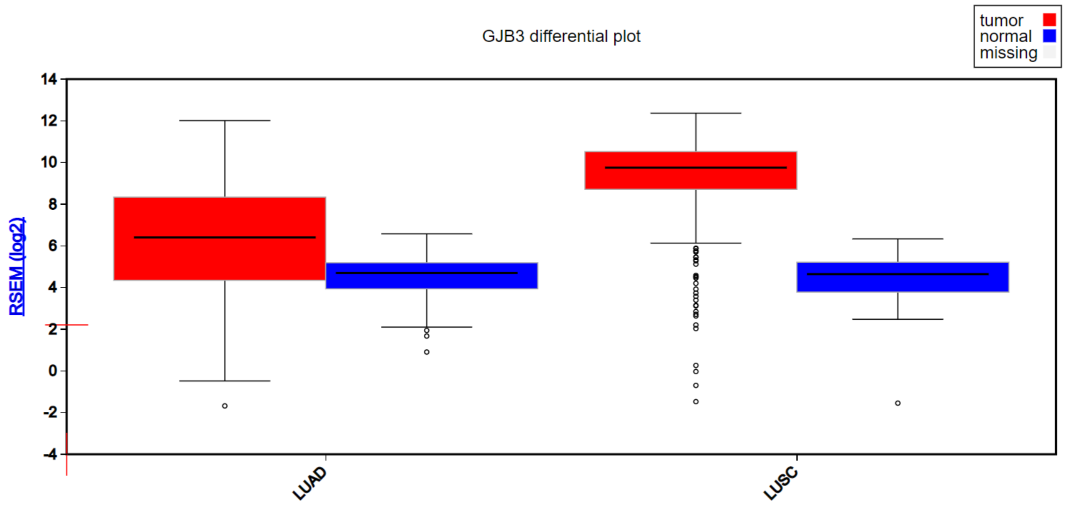
GJB3 differential plot
tumor
normal
missing
RSEM (log2)
14
12
10
8
6
4
2
0
-2
-4
LUAD
LUSC

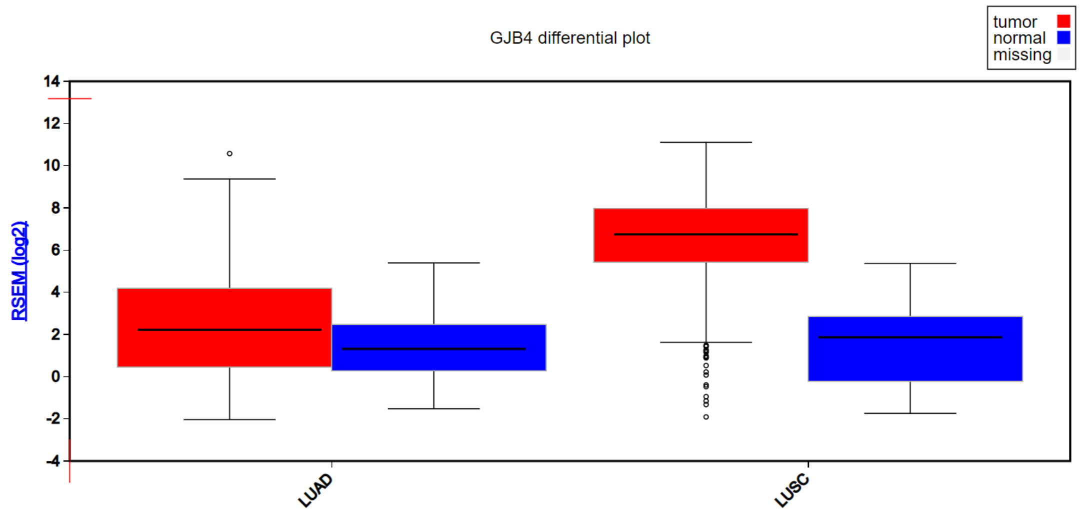

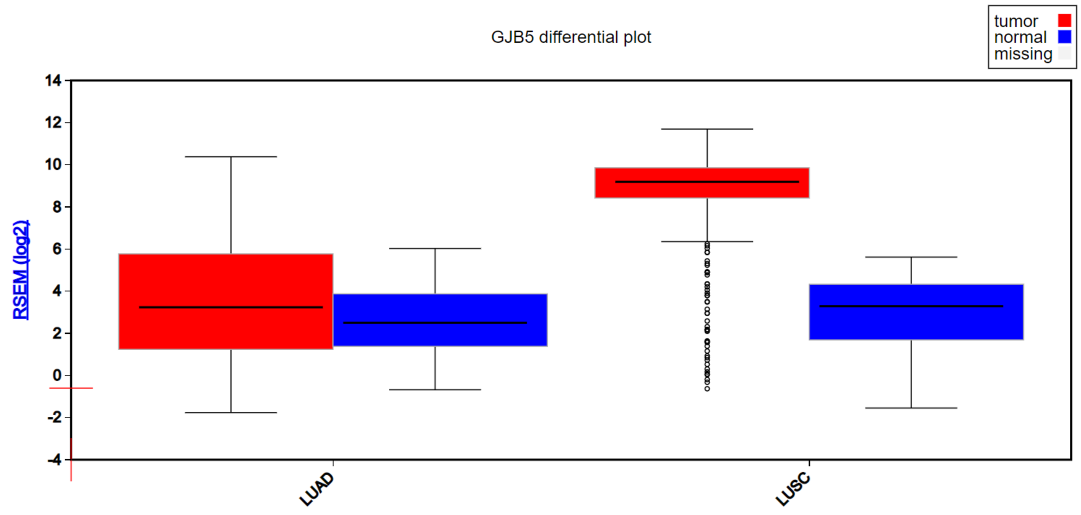

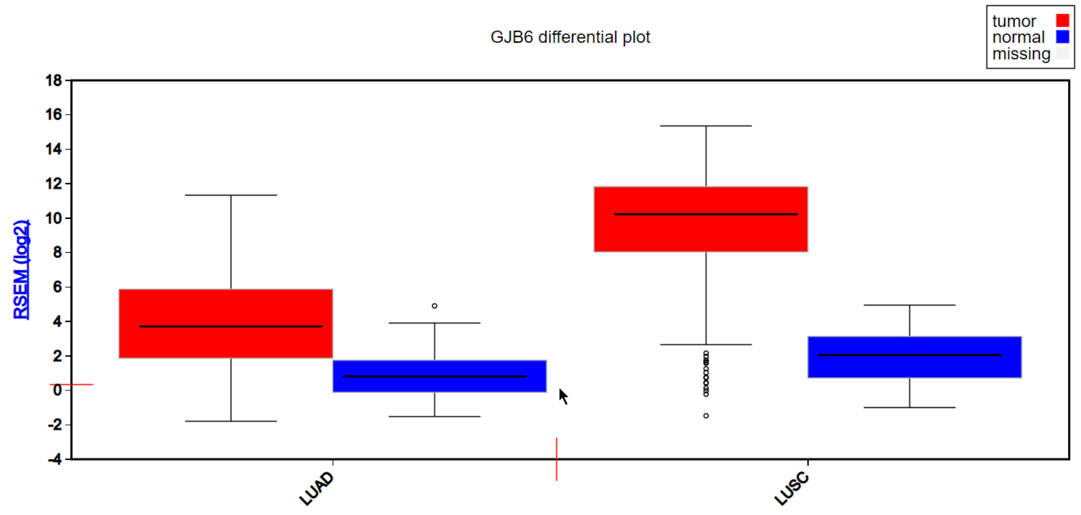

B) Decreased expression in both LUAD and LUSC tumours:

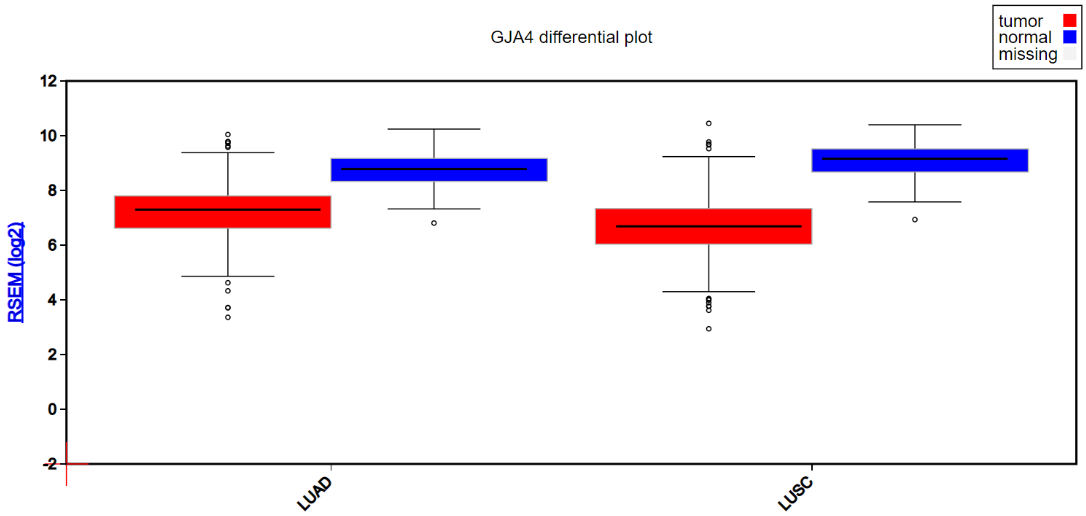
GJA4 differential plot
tumor
normal
missing
RSEM (log2)
12
10
8
6
4
2
0
-2
LUAD
LUSC

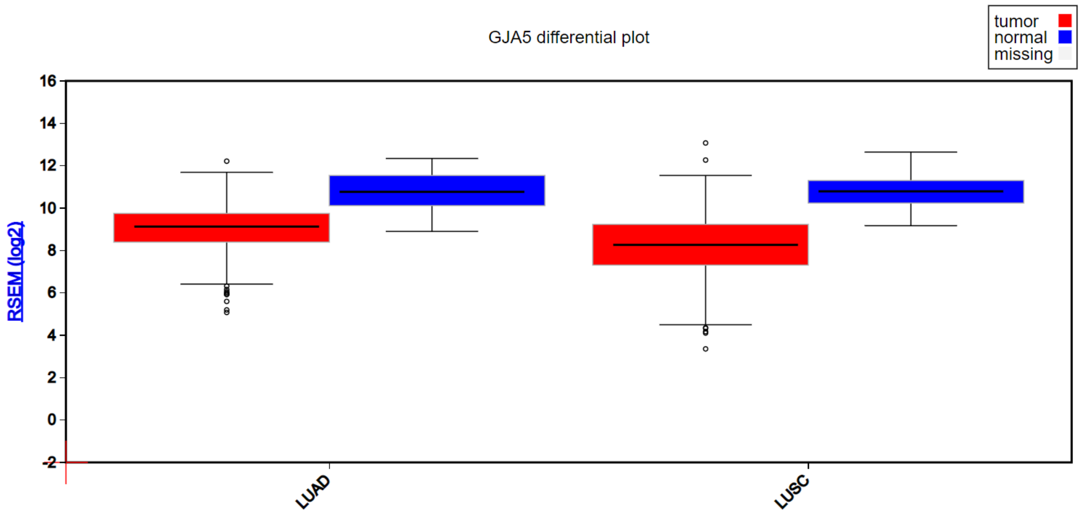
GJA5 differential plot
tumor
normal
missing
RSEM (log2)
16
14
12
10
8
6
4
2
0
-2
LUAD
LUSC

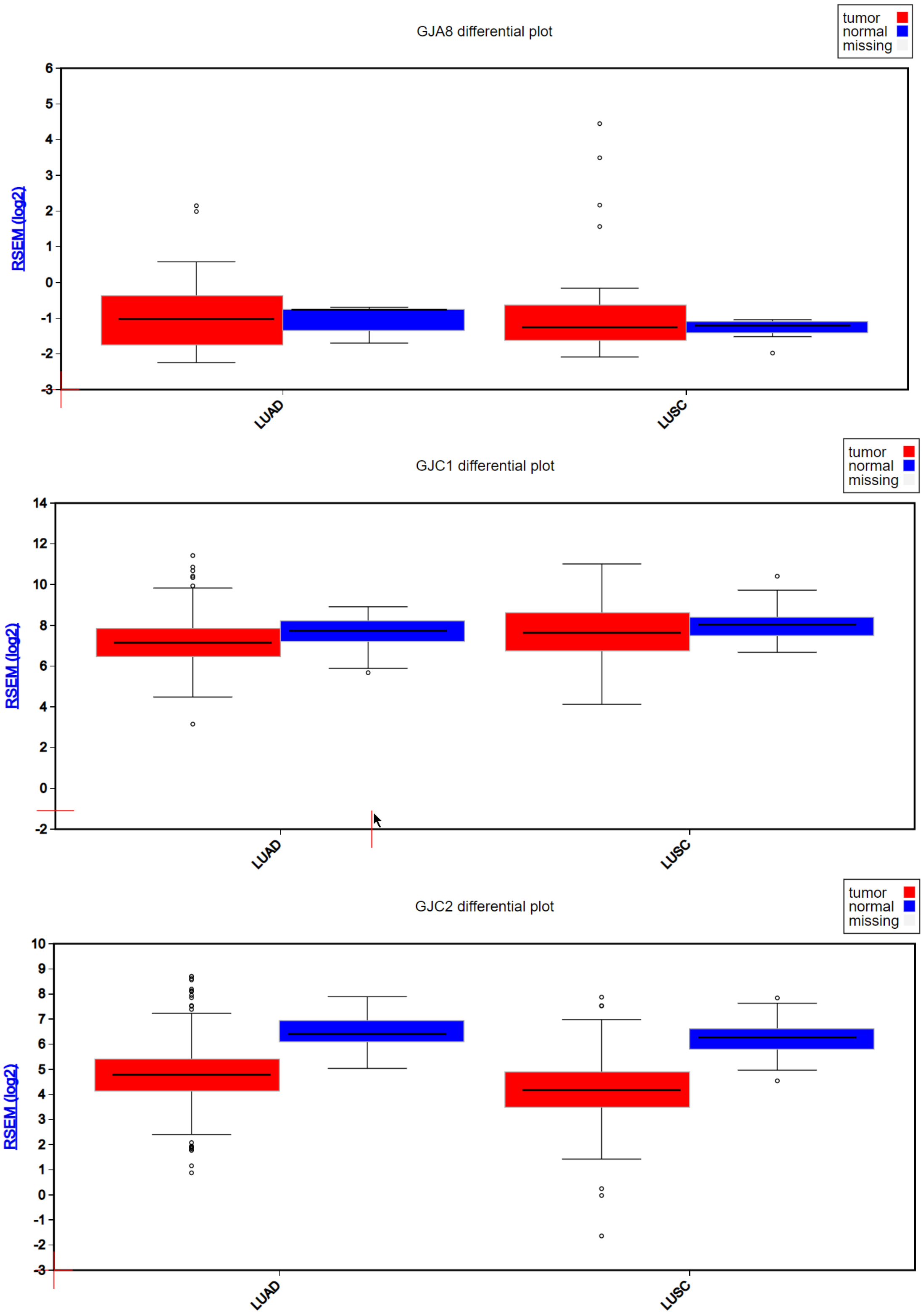
GJA8 differential plot
tumor
normal
missing
RSEM (log2)
6
5
4
3
2
1
0
-1
-2
-3
LUAD
LUSC
GJC1 differential plot
tumor
normal
missing
RSEM (log2)
14
12
10
8
6
4
2
0
-2
LUAD
LUSC
GJC2 differential plot
tumor
normal
missing
RSEM (log2)
10
9
8
7
6
5
4
3
2
1
0
-1
-2
-3
LUAD
LUSC

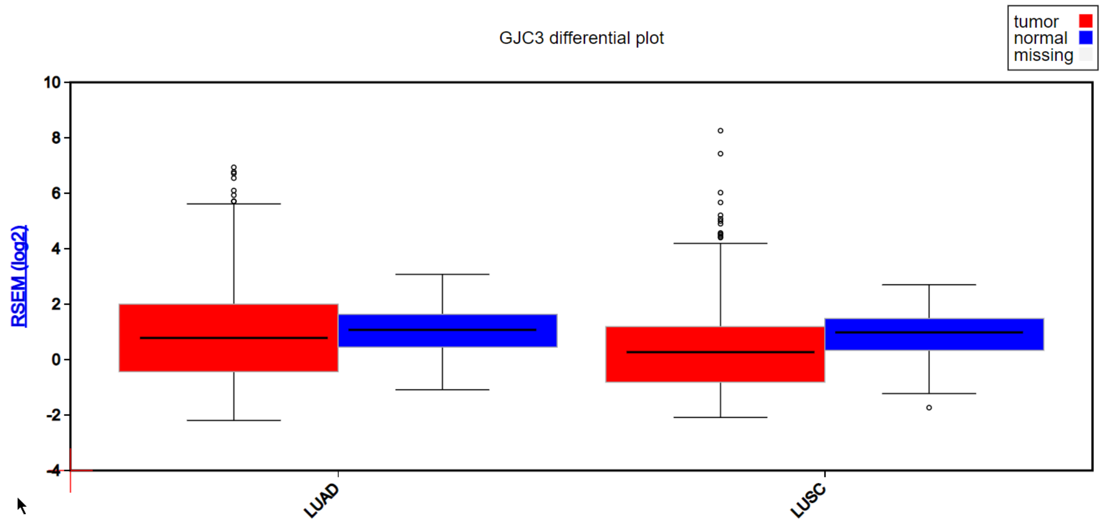
GJC3 differential plot
tumor
normal
missing
RSEM (log2)
10
8
6
4
2
0
-2
-4
LUAD
LUSC

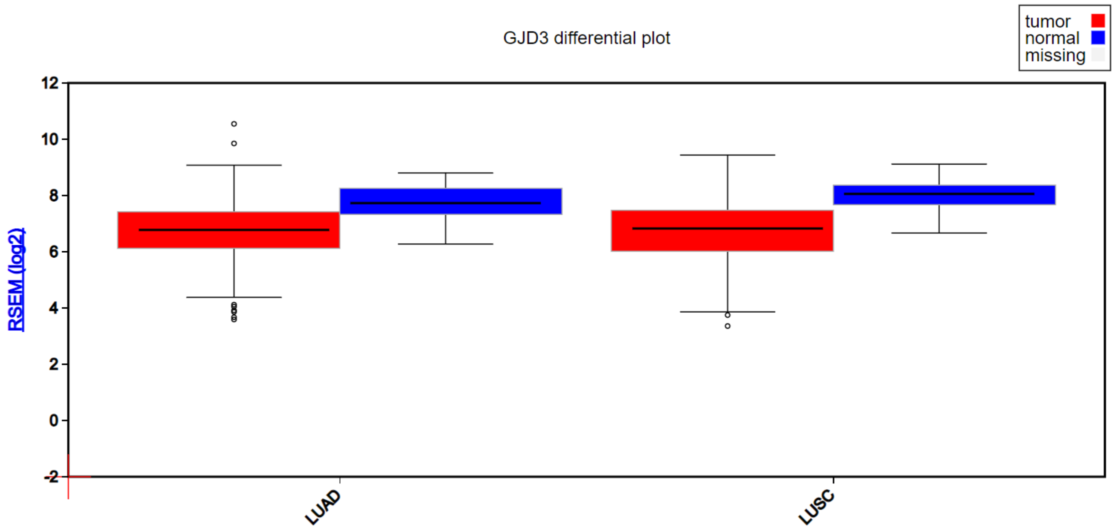
GJD3 differential plot
tumor
normal
missing
RSEM (log2)
12
10
8
6
4
2
0
-2
LUAD
LUSC

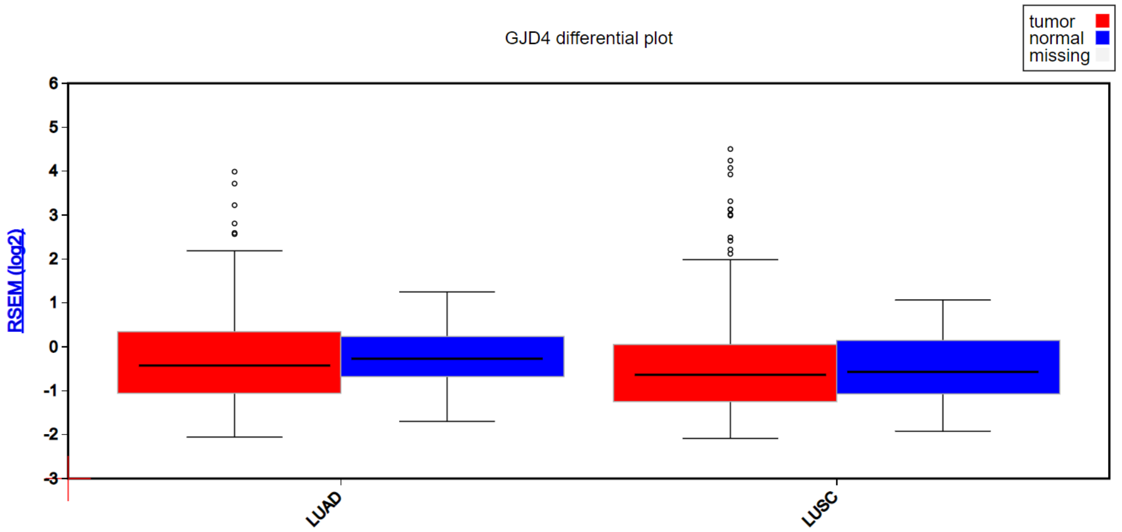
GJD4 differential plot
tumor
normal
missing
RSEM (log2)
6
5
4
3
2
1
0
-1
-2
-3
LUAD
LUSC

C) Mixed, increased and decreased expression in LUAD versus LUSC tumours:

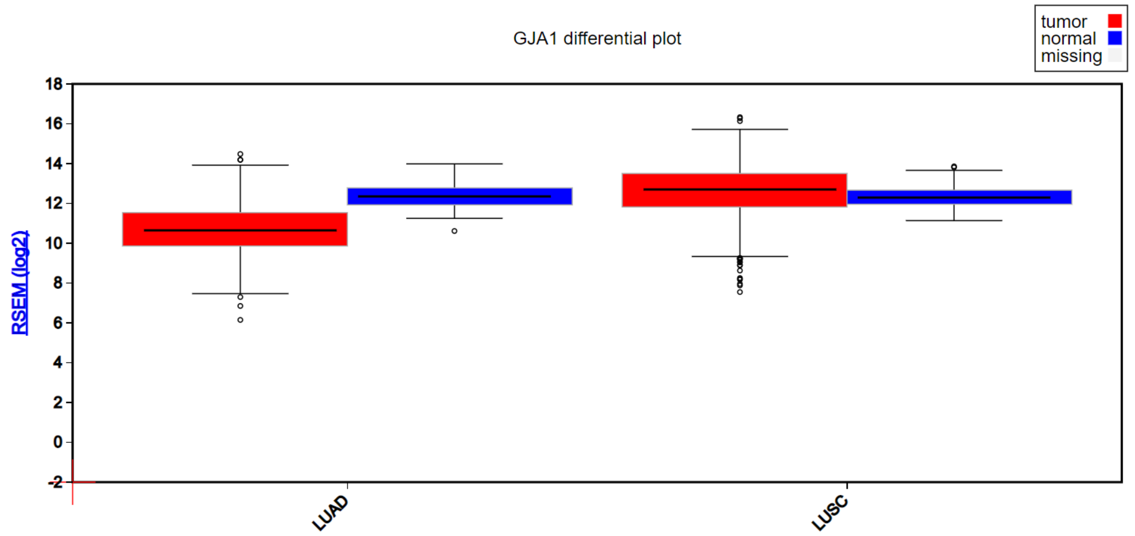

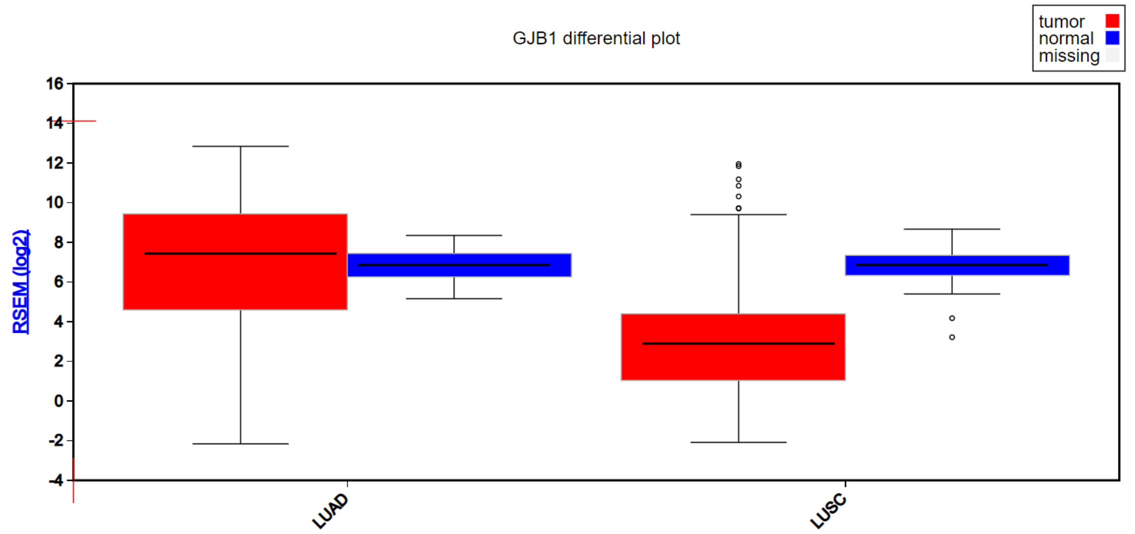

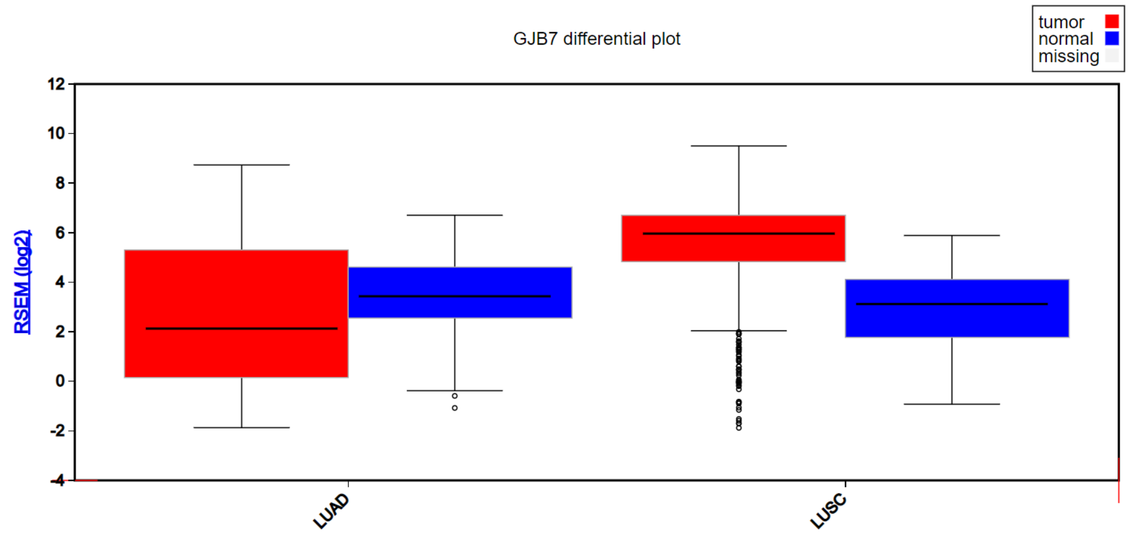
GJB7 differential plot
tumor
normal
missing
RSEM (log2)
12
10
8
6
4
2
0
-2
-4
LUAD
LUSC

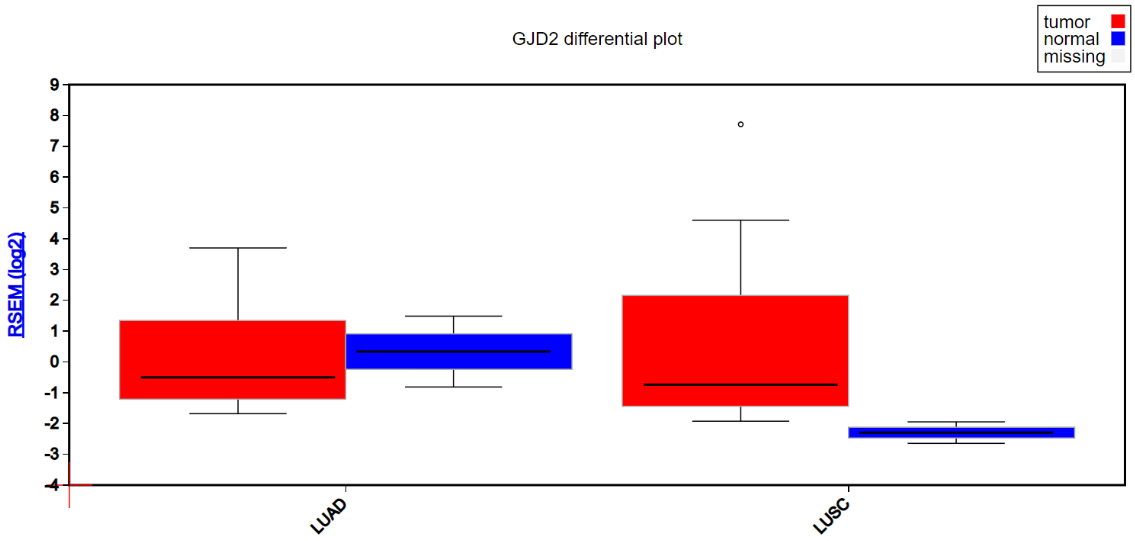
GJD2 differential plot
tumor
normal
missing
RSEM (log2)
9
8
7
6
5
4
3
2
1
0
-1
-2
-3
-4
LUAD
LUSC

Supplementary Figure S2.

Examples of methylation statuses of key connexin genes in healthy versus tumour tissue. A 450k Methylation Array displaying individual probes along the gene region is shown (equally distributed). Probes in green are part of the CpG cluster of this gene. Significant differences are highlighted with an asterisk on individual probes. These examples illustrate poor correlation for the four connexin genes in focus of this study (GJB1, GJB6, GJB2 and GJA1, all showing moderate or poor correlation) as well as GJB5 which is included as an example of a connexin gene which correlate moderately well between changes in methylation status and mRNA expression. See Figure 3 for a more detailed example of GJB2.

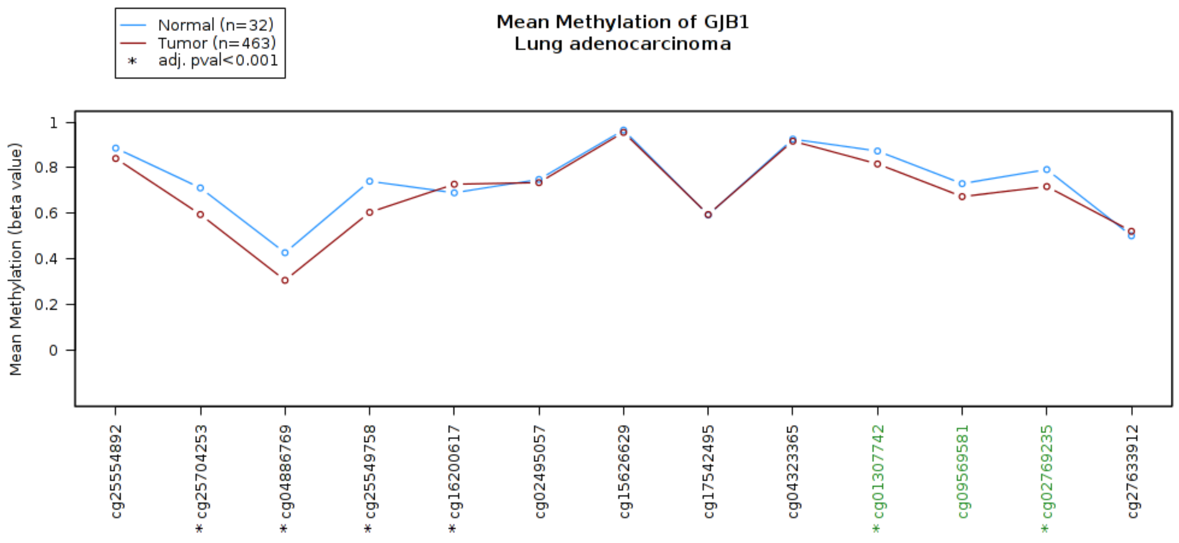

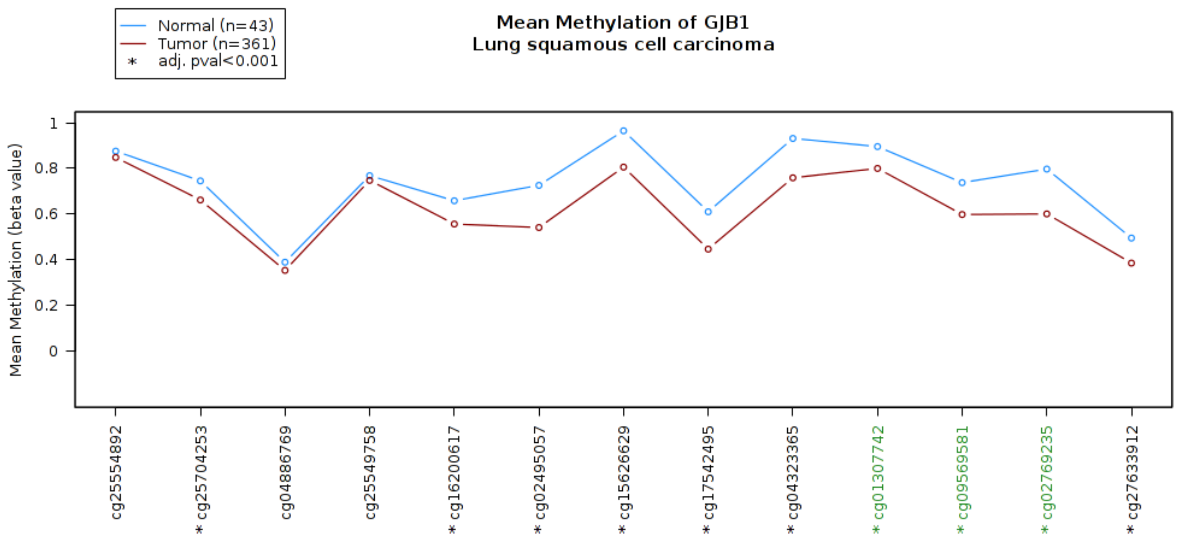

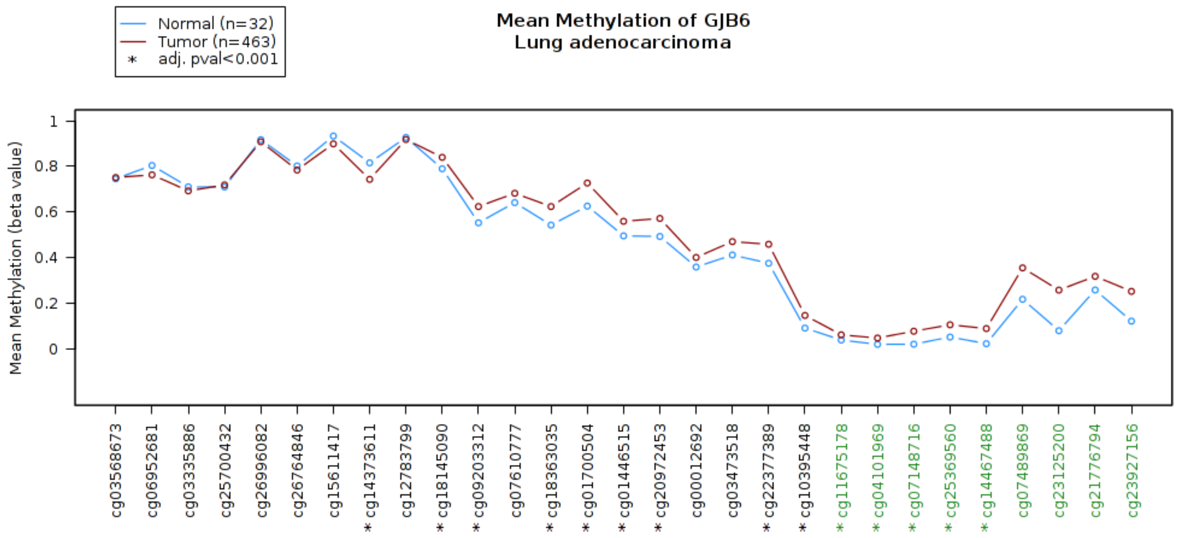
Normal (n=32)
Tumor (n=463)
* adj. pval<0.001
Mean Methylation of GJB6
Lung adenocarcinoma
Mean Methylation (beta value)
1
0.8
0.6
0.4
0.2
0
cg03568673
cg06952681
cg03335886
cg25700432
cg26996082
cg26764846
cg15611417
* cg14373611
cg12783799
* cg18145090
* cg09203312
cg07610777
* cg18363035
* cg01700504
* cg01446515
* cg20972453
cg00012692
cg03473518
* cg22377389
* cg10395448
* cg11675178
* cg04101969
* cg07148716
* cg25369560
* cg14467488
cg07489869
cg23125200
cg21776794
cg23927156

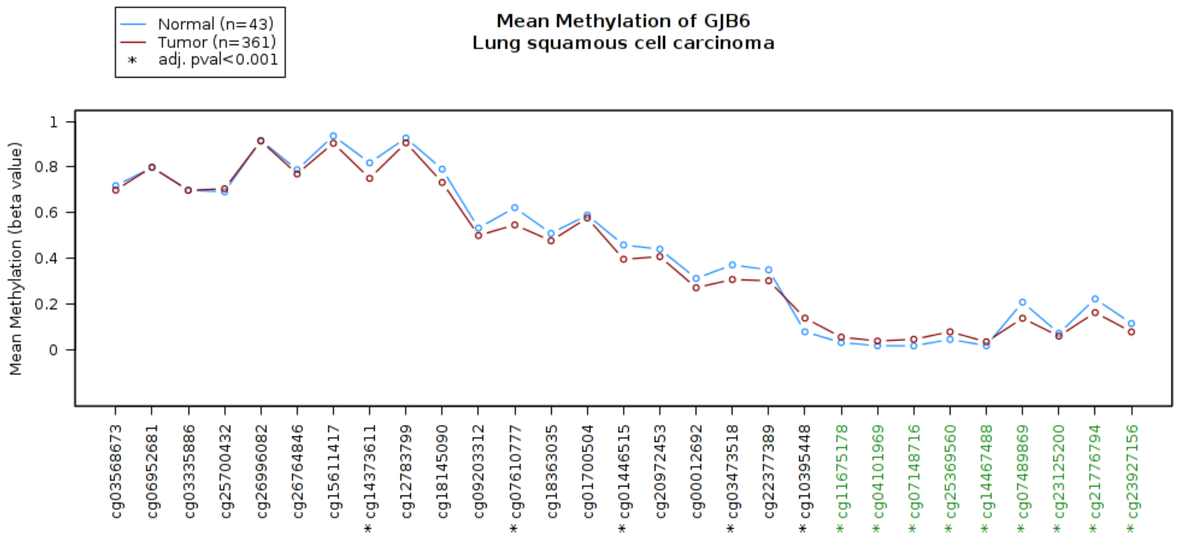
Normal (n=43)
Tumor (n=361)
* adj. pval<0.001
Mean Methylation of GJB6
Lung squamous cell carcinoma
Mean Methylation (beta value)
1
0.8
0.6
0.4
0.2
0
cg03568673
cg06952681
cg03335886
cg25700432
cg26996082
cg26764846
cg15611417
* cg14373611
cg12783799
cg18145090
cg09203312
* cg07610777
cg18363035
cg01700504
* cg01446515
cg20972453
cg00012692
* cg03473518
cg22377389
* cg10395448
* cg11675178
* cg04101969
* cg07148716
* cg25369560
* cg14467488
* cg07489869
* cg23125200
* cg21776794
* cg23927156

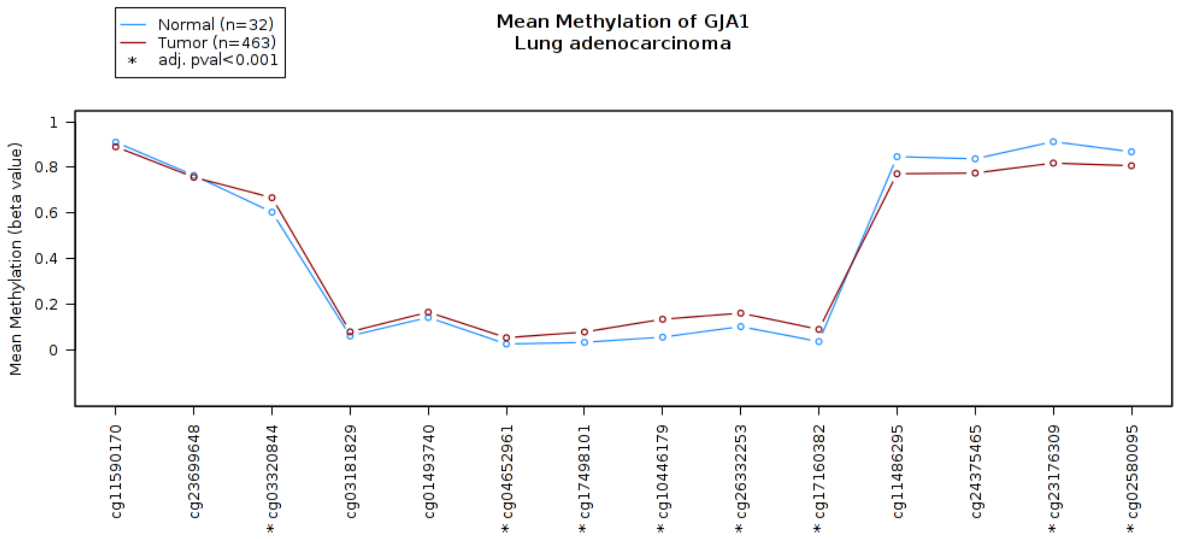
Normal (n=32)
Tumor (n=463)
* adj. pval<0.001
Mean Methylation of GJA1
Lung adenocarcinoma
Mean Methylation (beta value)
1
0.8
0.6
0.4
0.2
0
cg11590170
cg23699648
* cg03320844
cg03181829
cg01493740
* cg04652961
* cg17498101
* cg10446179
* cg26332253
* cg17160382
cg11486295
cg24375465
* cg23176309
* cg02580095

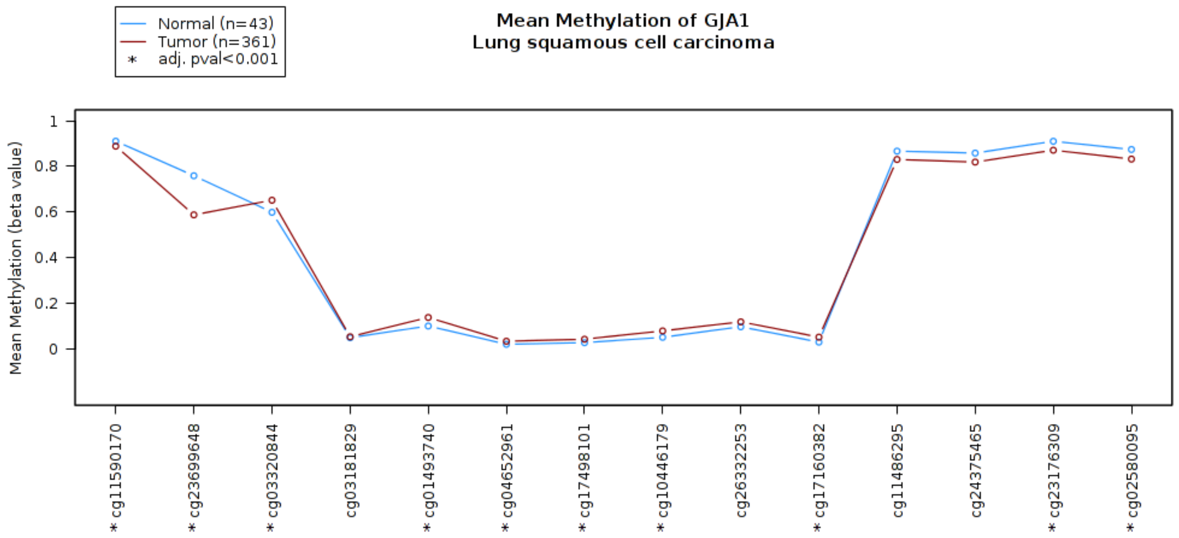
Normal (n=43)
Tumor (n=361)
* adj. pval<0.001
Mean Methylation of GJA1
Lung squamous cell carcinoma
Mean Methylation (beta value)
1
0.8
0.6
0.4
0.2
0
* cg11590170
* cg23699648
* cg03320844
cg03181829
* cg01493740
* cg04652961
* cg17498101
* cg10446179
cg26332253
* cg17160382
cg11486295
cg24375465
* cg23176309
* cg02580095

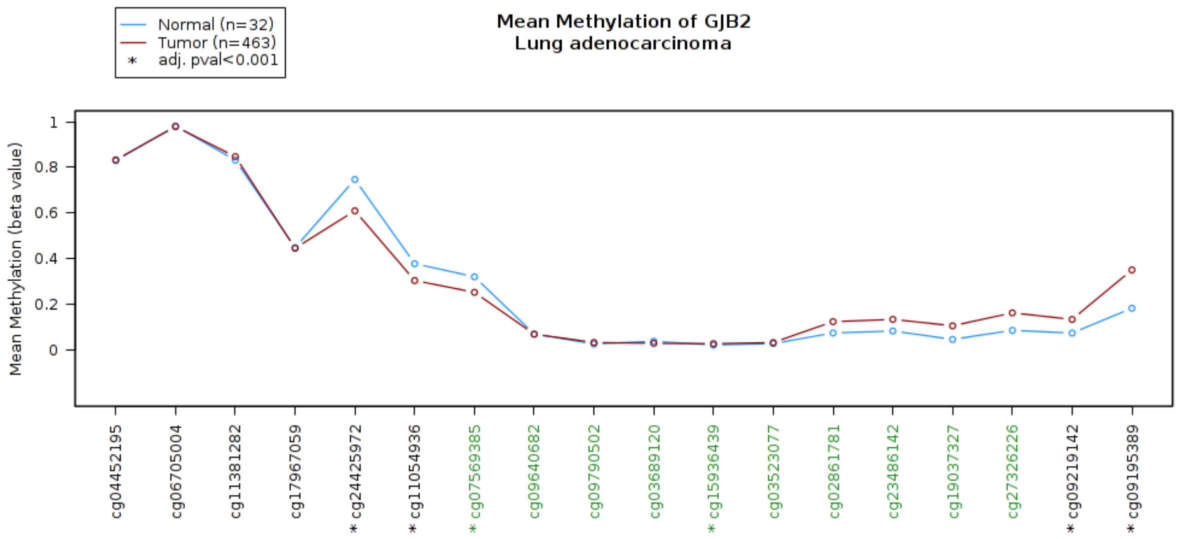
Normal (n=32)
Tumor (n=463)
* adj. pval<0.001
Mean Methylation of GJB2
Lung adenocarcinoma
Mean Methylation (beta value)
1
0.8
0.6
0.4
0.2
0
cg04452195
cg06705004
cg11381282
cg17967059
* cg24425972
* cg11054936
* cg07569385
cg09640682
cg09790502
cg03689120
* cg15936439
cg03523077
cg02861781
cg23486142
cg19037327
cg27326226
* cg09219142
* cg09195389

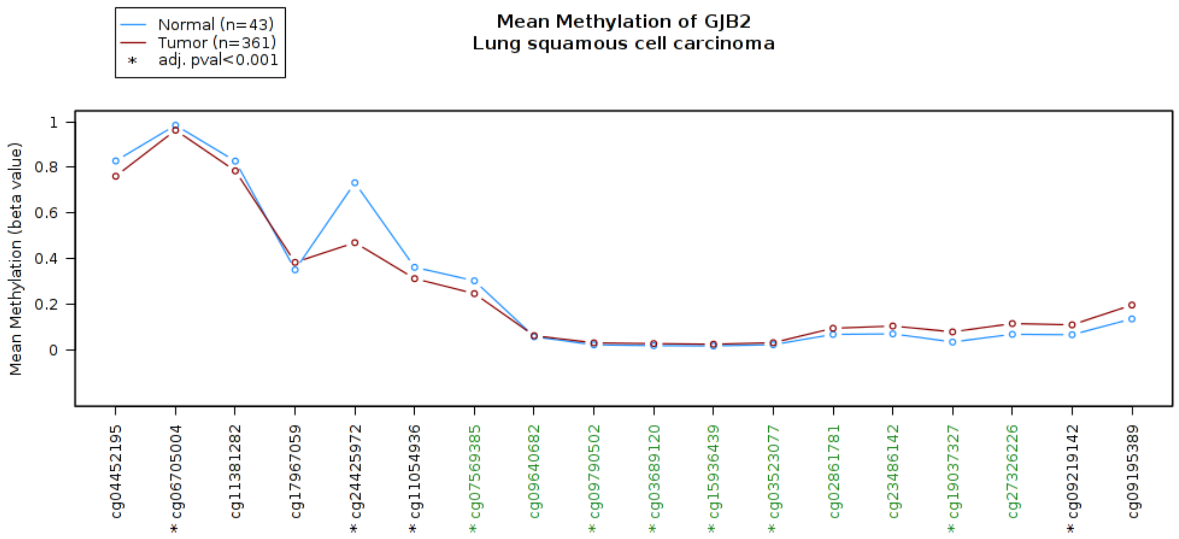
Normal (n=43)
Tumor (n=361)
* adj. pval<0.001
Mean Methylation of GJB2
Lung squamous cell carcinoma
Mean Methylation (beta value)
1
0.8
0.6
0.4
0.2
0
cg04452195
* cg06705004
cg11381282
cg17967059
* cg24425972
* cg11054936
* cg07569385
cg09640682
* cg09790502
* cg03689120
* cg15936439
* cg03523077
cg02861781
cg23486142
* cg19037327
cg27326226
* cg09219142
cg09195389

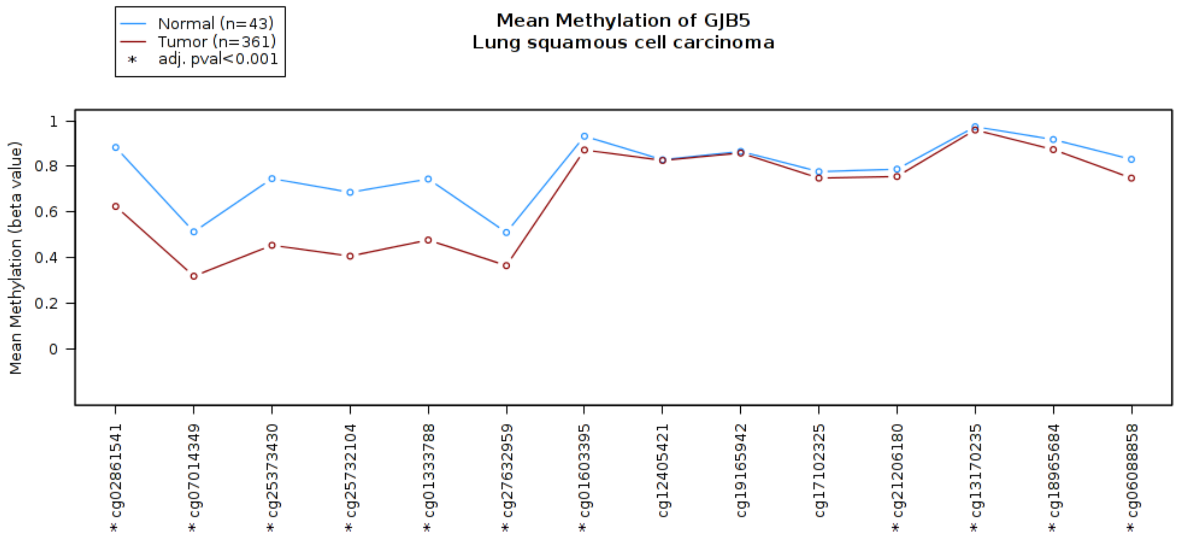

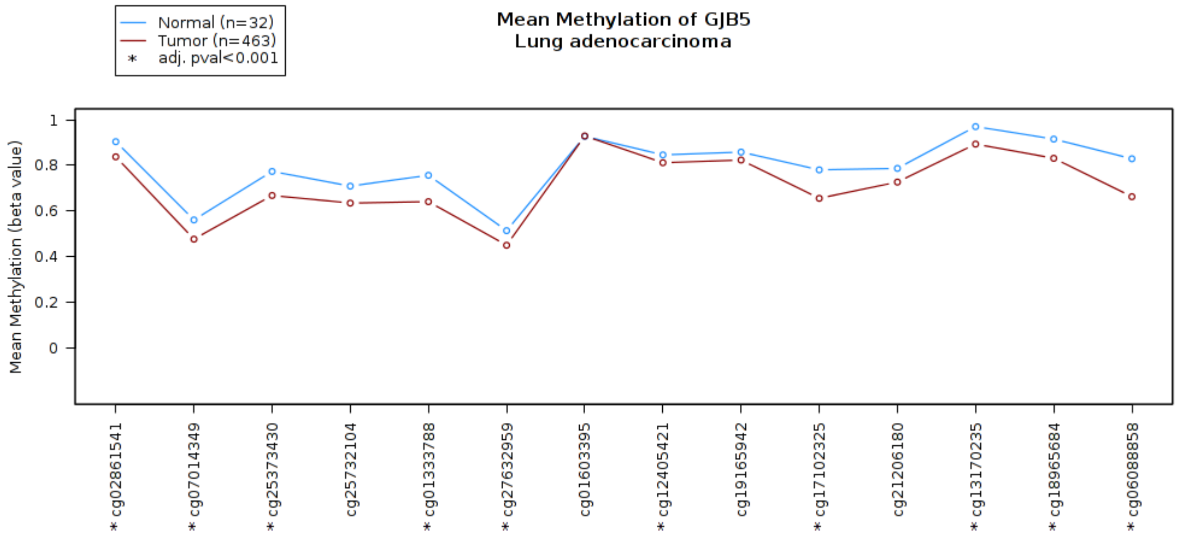

Supplementary Figure S3.

Kaplan-Meier curves of connexins known to drive lung tumorigenesis. The cohort expressing high levels of connexin mRNA is depicted in red, and the cohort with low connexin levels is in black. Probability refers to the likelihood of being alive at any time point. A hazard ratio (HR) below 1 suggests an association with better prognosis, whereas a HR above 1 suggests better prognosis (in the cohort expressing high levels of connexins).

	**NSCLC**	**LUSC**	**LUAD**
**Cx46**<br><br>***GJA3***	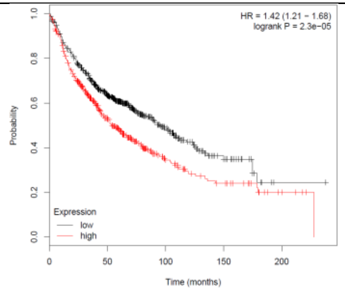	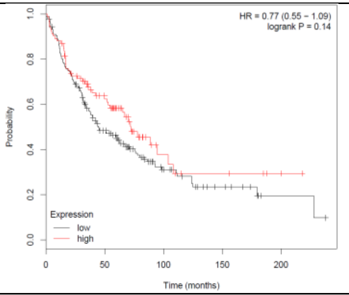	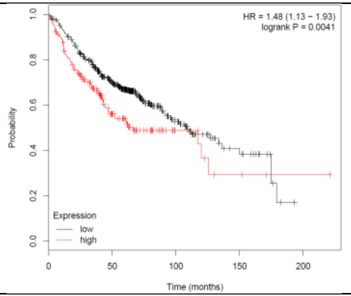

Cx37 *GJA4*

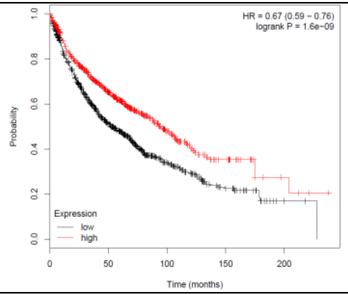

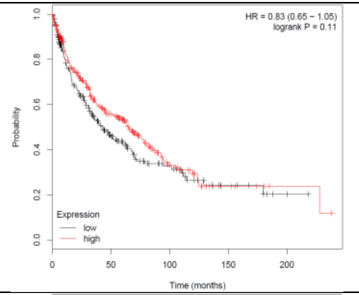

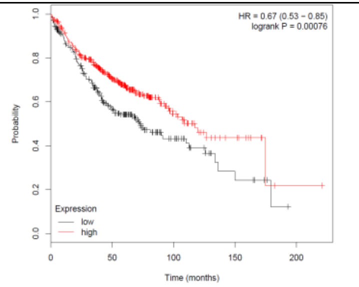

Cx40 *GJA5*

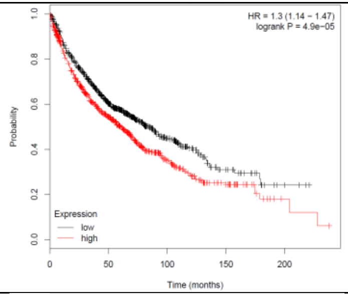

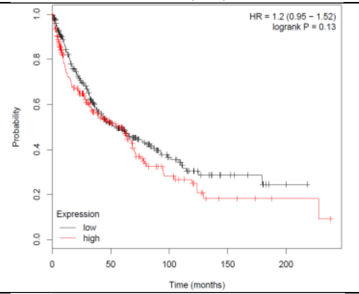

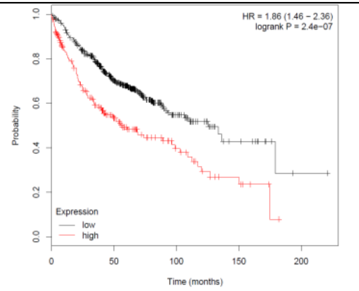

Cx50 *GJA8*

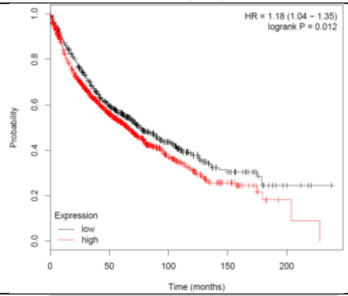

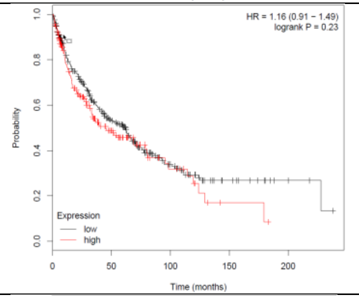

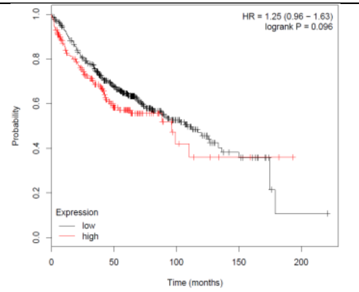

Cx62 *GJA10*

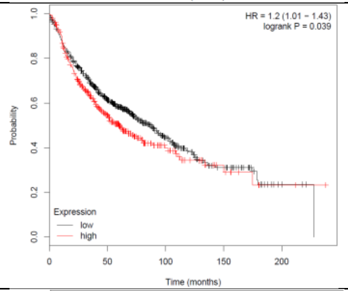

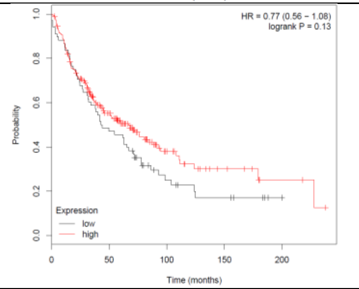

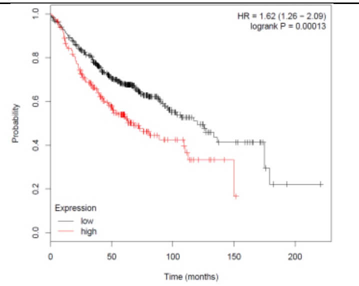

Cx31 *GJB3*

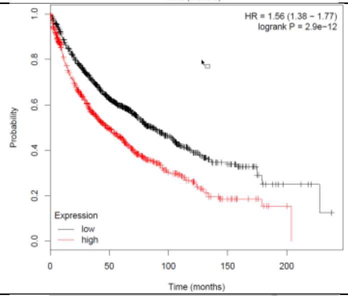

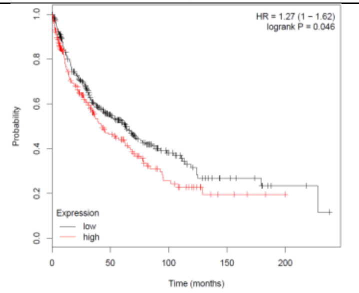

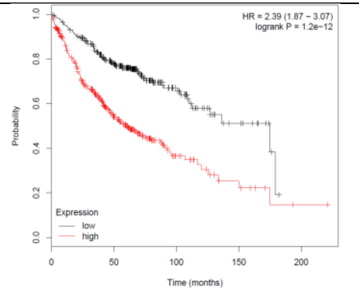

Cx31.1 *GJB5*

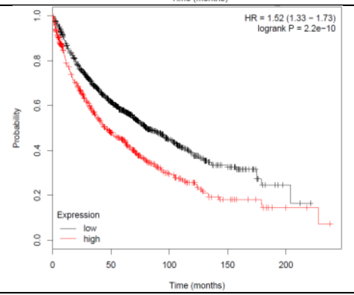

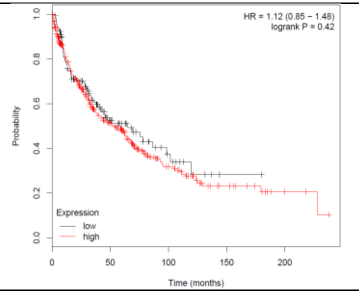

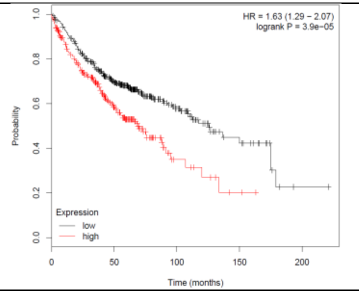

**Cx45**<br>***GJC1***	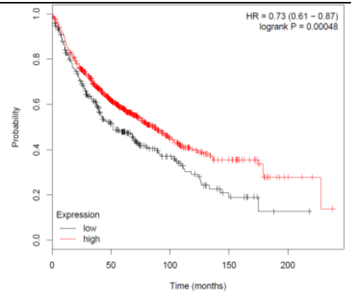	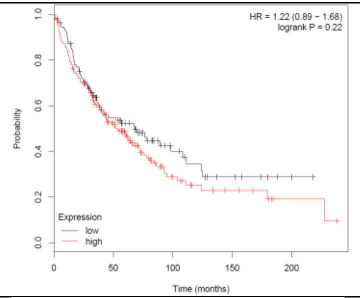	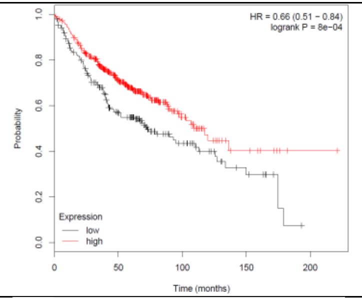
**Cx47**<br>***GJC2***	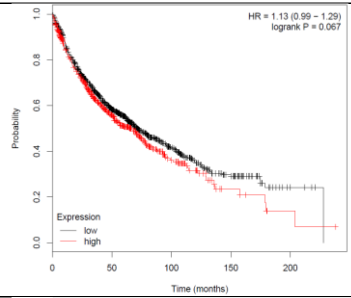	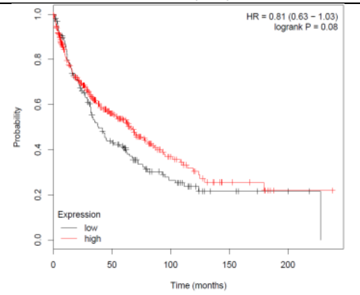	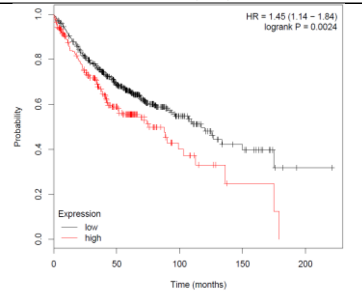
**Cx30.2**<br>***GJC3***	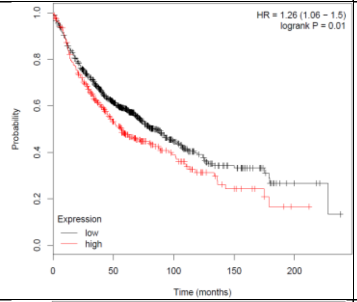	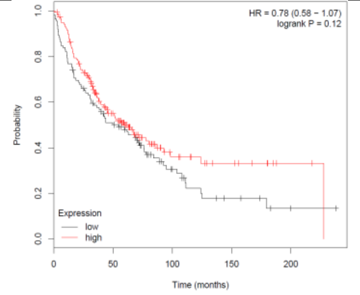	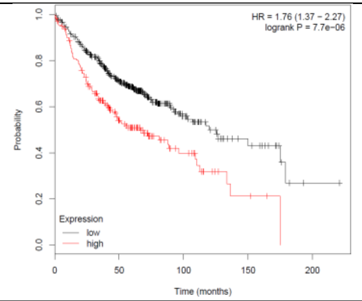
**Cx36**<br>***GJD2***	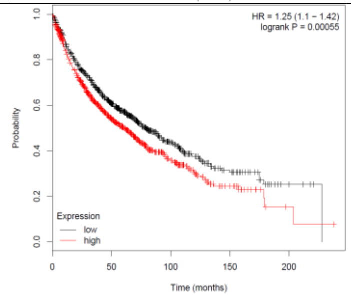	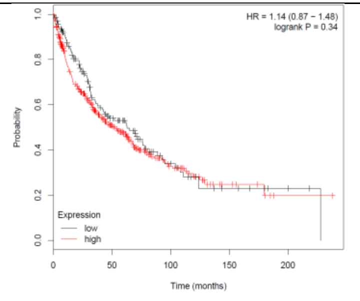	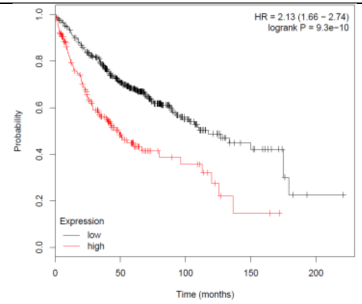
**Cx31.9**<br>***GJD3***	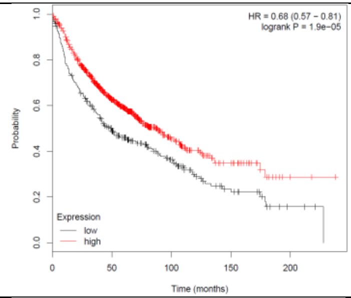	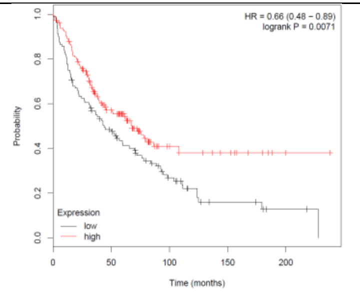	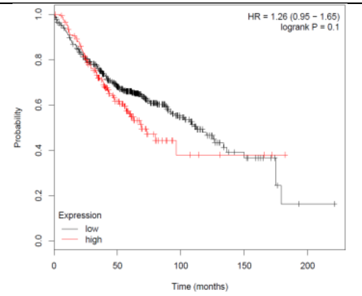
**Cx40.1**<br>***GJD4***	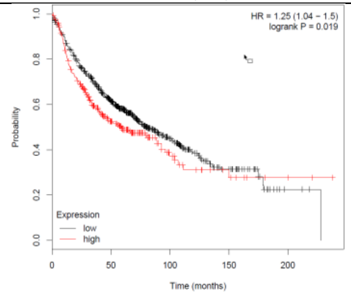	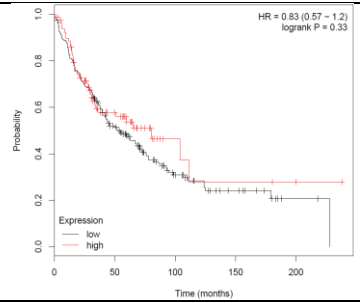	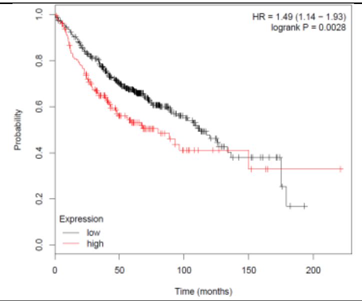

Supplementary Figure S4.

Kaplan-Meier curves based on Cx43 expression patterns detected by immunohistochemistry of lung cancer samples. Tumours with IHC deemed significant are depicted in red and compared with the rest of the tumour samples (in blue). *p*-values refer to the log-rank test (Mantel-Cox analysis). Average survival times of the patient cohorts are listed.

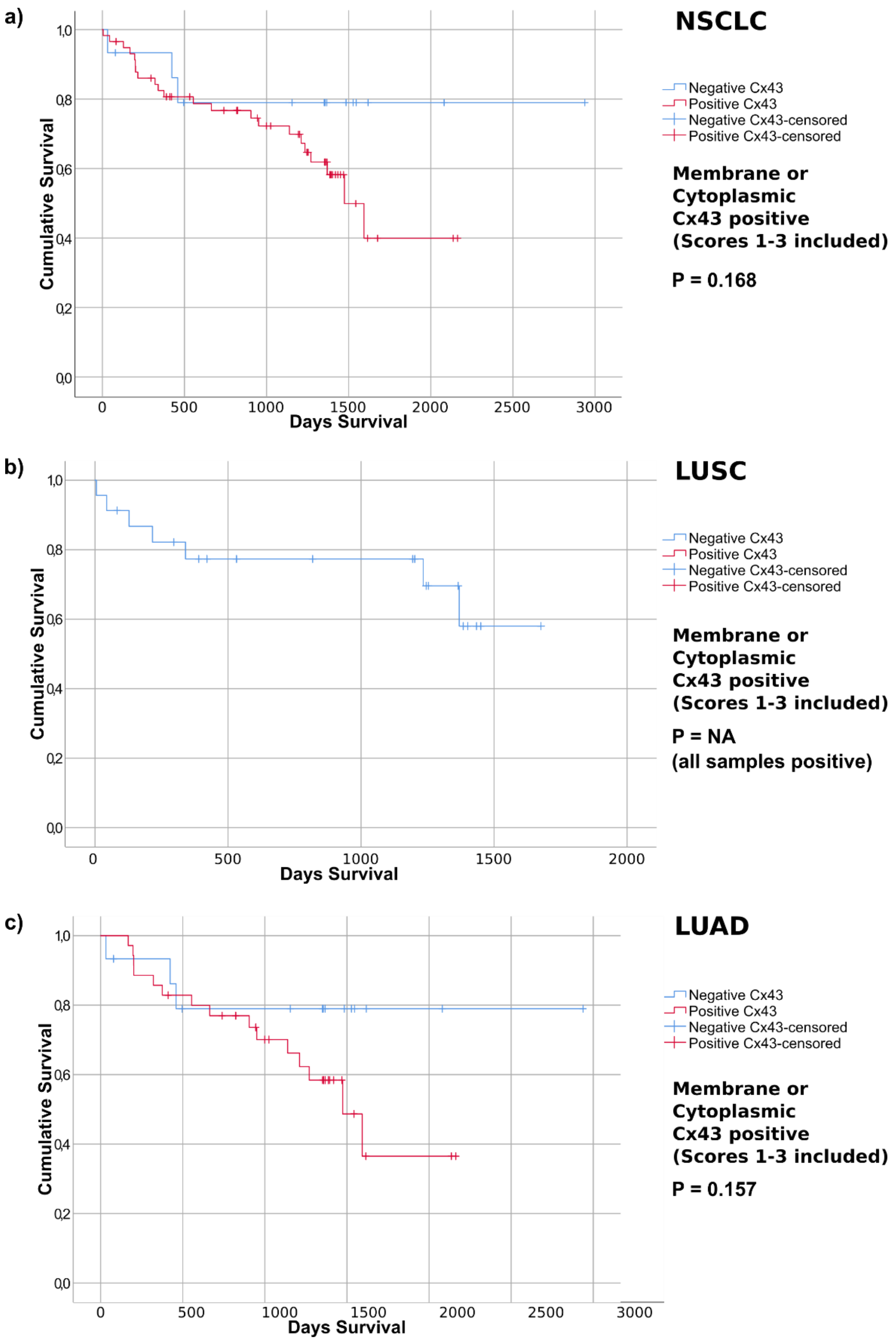

d)

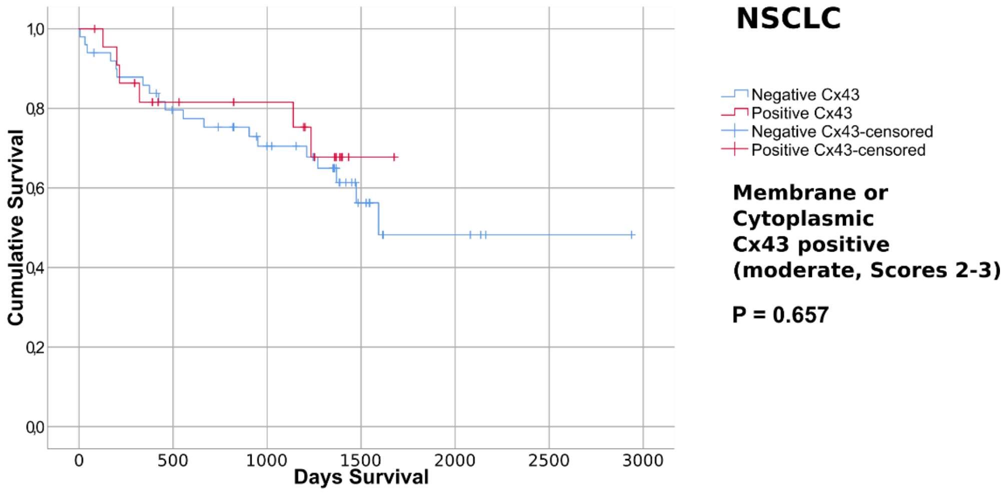
NSCLC
Negative Cx43
Positive Cx43
Negative Cx43-censored
Positive Cx43-censored
Membrane or Cytoplasmic Cx43 positive (moderate, Scores 2-3)
P = 0.657
Cumulative Survival
1,0
0,8
0,6
0,4
0,2
0,0
0
500
1000
1500
2000
2500
3000
Days Survival

e)

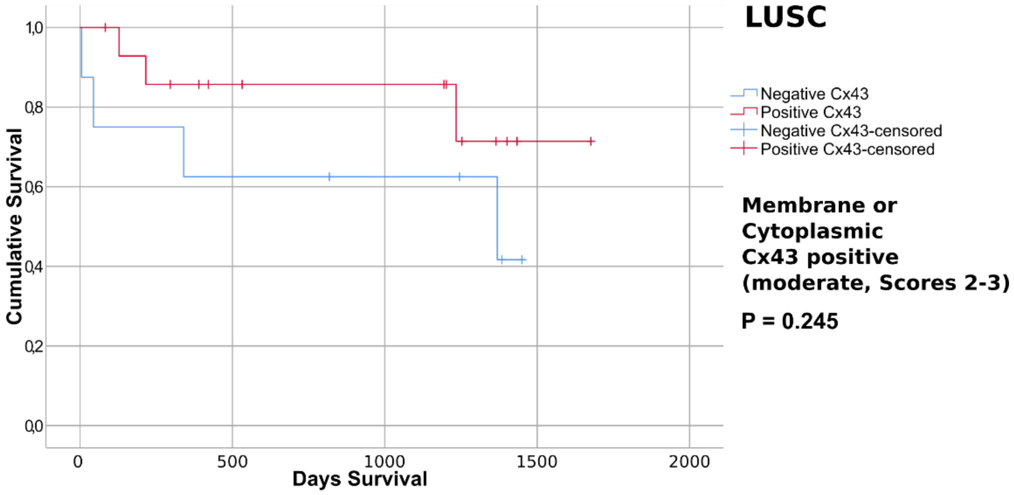
LUSC
Negative Cx43
Positive Cx43
Negative Cx43-censored
Positive Cx43-censored
Membrane or Cytoplasmic Cx43 positive (moderate, Scores 2-3)
P = 0.245
Cumulative Survival
1,0
0,8
0,6
0,4
0,2
0,0
0
500
1000
1500
2000
Days Survival

f)

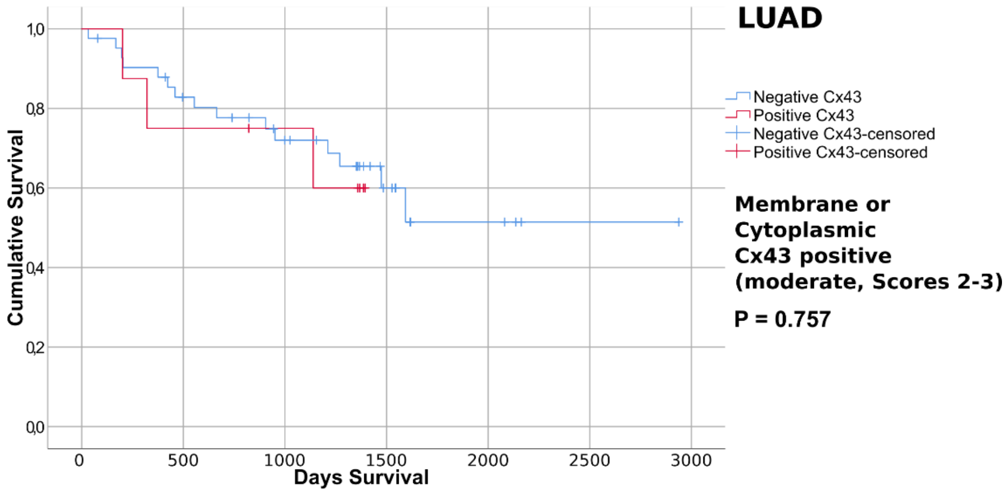
LUAD
Negative Cx43
Positive Cx43
Negative Cx43-censored
Positive Cx43-censored
Membrane or Cytoplasmic Cx43 positive (moderate, Scores 2-3)
P = 0.757
Cumulative Survival
1,0
0,8
0,6
0,4
0,2
0,0
0
500
1000
1500
2000
2500
3000
Days Survival

g)

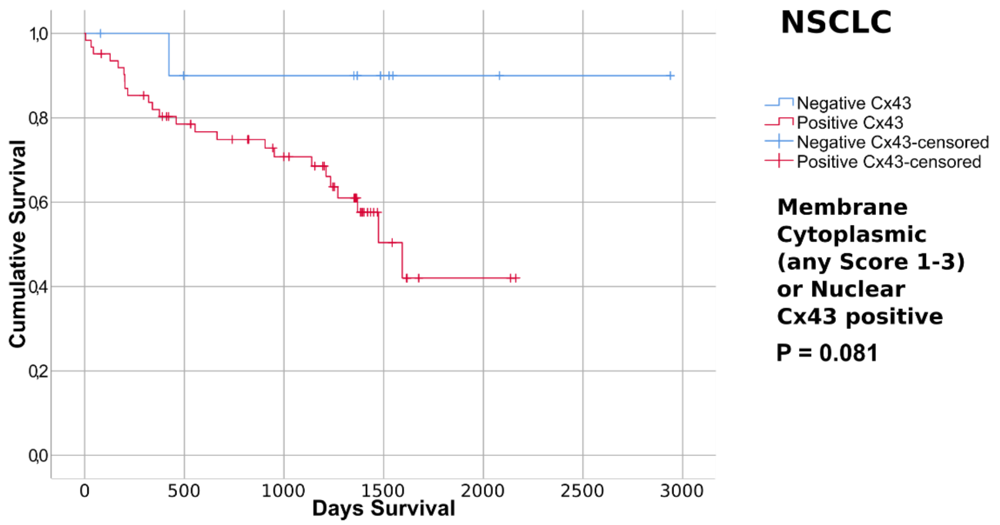
NSCLC
Cumulative Survival
1,0
0,8
0,6
0,4
0,2
0,0
0
500
1000
1500
2000
2500
3000
Days Survival
Negative Cx43
Positive Cx43
Negative Cx43-censored
Positive Cx43-censored
Membrane Cytoplasmic (any Score 1-3) or Nuclear Cx43 positive
P = 0.081

h)

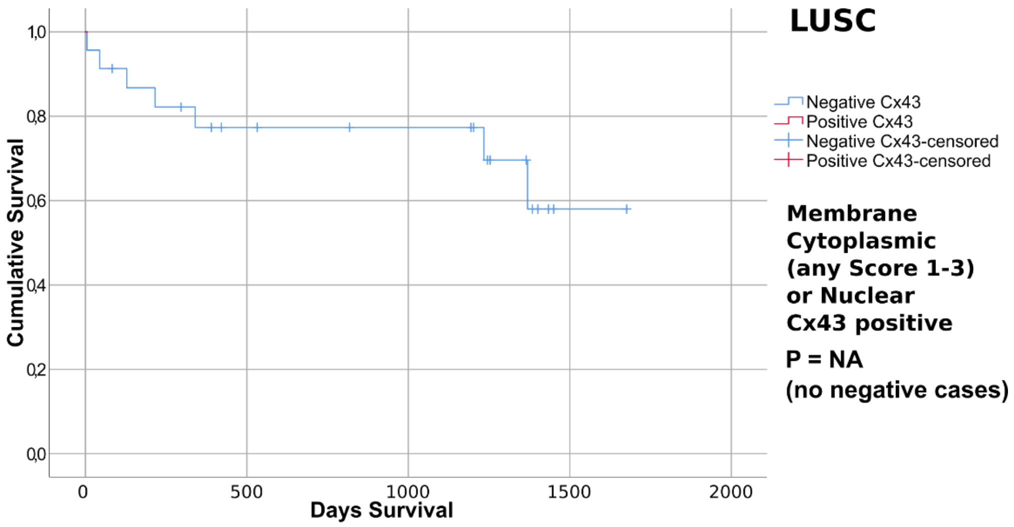
LUSC
Cumulative Survival
1,0
0,8
0,6
0,4
0,2
0,0
0
500
1000
1500
2000
Days Survival
Negative Cx43
Positive Cx43
Negative Cx43-censored
Positive Cx43-censored
Membrane Cytoplasmic (any Score 1-3) or Nuclear Cx43 positive
P = NA
(no negative cases)

i)

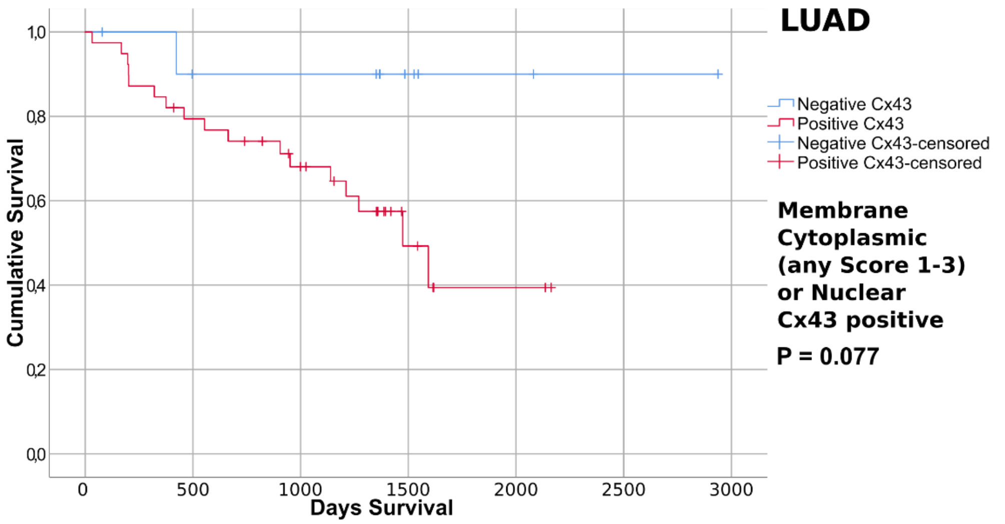
LUAD
Cumulative Survival
1,0
0,8
0,6
0,4
0,2
0,0
0
500
1000
1500
2000
2500
3000
Days Survival
Negative Cx43
Positive Cx43
Negative Cx43-censored
Positive Cx43-censored
Membrane Cytoplasmic (any Score 1-3) or Nuclear Cx43 positive
P = 0.077

j)

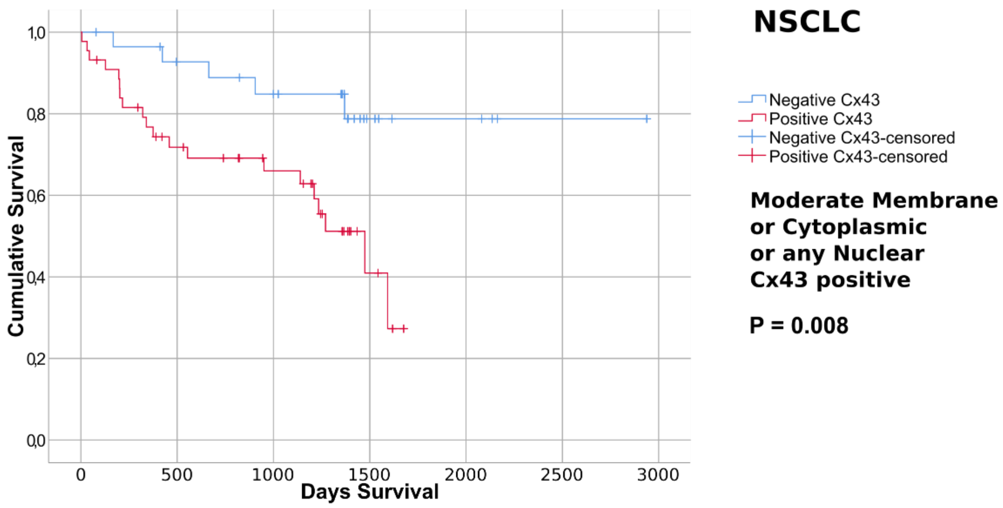
NSCLC
Negative Cx43
Positive Cx43
Negative Cx43-censored
Positive Cx43-censored
Moderate Membrane or Cytoplasmic or any Nuclear Cx43 positive
P = 0.008
Cumulative Survival
Days Survival
1,0
0,8
0,6
0,4
0,2
0,0
0
500
1000
1500
2000
2500
3000

k)

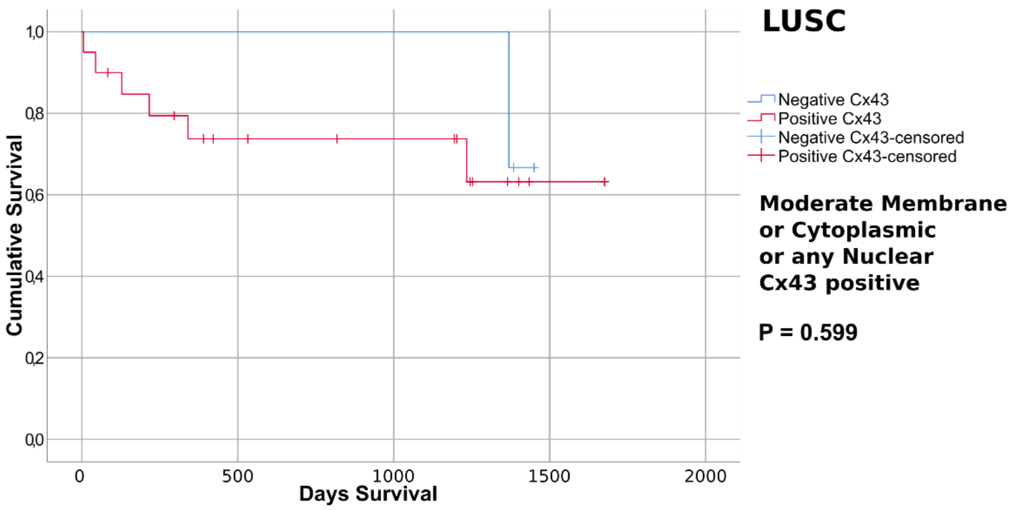
LUSC
Negative Cx43
Positive Cx43
Negative Cx43-censored
Positive Cx43-censored
Moderate Membrane or Cytoplasmic or any Nuclear Cx43 positive
P = 0.599
Cumulative Survival
Days Survival
1,0
0,8
0,6
0,4
0,2
0,0
0
500
1000
1500
2000

l)

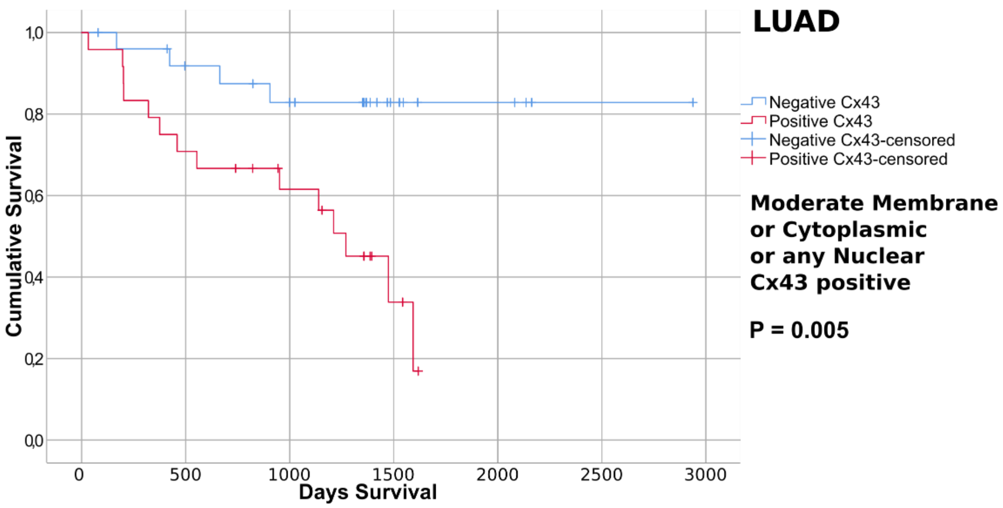
LUAD
Negative Cx43
Positive Cx43
Negative Cx43-censored
Positive Cx43-censored
Moderate Membrane or Cytoplasmic or any Nuclear Cx43 positive
P = 0.005
Cumulative Survival
Days Survival
1,0
0,8
0,6
0,4
0,2
0,0
0
500
1000
1500
2000
2500
3000
